# Simple and efficient differentiation of human iPSCs into contractible skeletal muscles for muscular disease modeling

**DOI:** 10.1038/s41598-023-34445-9

**Published:** 2023-05-25

**Authors:** Muhammad Irfanur Rashid, Takuji Ito, Fuyuki Miya, Daisuke Shimojo, Kanae Arimoto, Kazunari Onodera, Rina Okada, Takunori Nagashima, Kazuki Yamamoto, Zohora Khatun, Rayhanul Islam Shimul, Jun-ichi Niwa, Masahisa Katsuno, Gen Sobue, Hideyuki Okano, Hidetoshi Sakurai, Kazunori Shimizu, Manabu Doyu, Yohei Okada

**Affiliations:** 1 Department of Neural iPSC Research, Institute for Medical Science of Aging, Aichi Medical University, 1-1 Yazakokarimata, Nagakute, Aichi 480-1195 Japan; 2 Department of Neurology, Aichi Medical University School of Medicine, 1-1 Yazakokarimata, Nagakute, Aichi 480-1195 Japan; 3 Japan Society for the Promotion of Science, 5-3-1 Kojimachi, Chiyoda-ku, Tokyo, 102-0083 Japan; 4 Center for Medical Genetics, Keio University School of Medicine, 35 Shinanomachi, Shinjuku-ku, Tokyo, 160-8582 Japan; 5 Department of Physiology, Keio University School of Medicine, 35 Shinanomachi, Shinjuku-ku, Tokyo, 160-8582 Japan; 6 Department of Biomolecular Engineering, Graduate School of Engineering, Nagoya University, Furo-cho, Chikusa-ku, Nagoya, Aichi 464-8603 Japan; 7 Department of Neurology, Nagoya University Graduate School of Medicine, Showa-ku, Nagoya, Aichi 466-8650 Japan; 8 Department of Clinical Research Education, Nagoya University Graduate School of Medicine, Showa-ku, Nagoya, Aichi 466-8650 Japan; 9 Aichi Medical University, 1-1 Yazakokarimata, Nagakute, Aichi 480-1195 Japan; 10 Department of Clinical Application, Center for iPS Cell Research and Application (CiRA), Kyoto University, 53 Kawahara-cho, Shogoin, Sakyo-ku, Kyoto, 606-8507 Japan

**Keywords:** Neuroscience, Stem cells, Pluripotent stem cells, Stem-cell differentiation

## Abstract

Pathophysiological analysis and drug discovery targeting human diseases require disease models that suitably recapitulate patient pathology. Disease-specific human induced pluripotent stem cells (hiPSCs) differentiated into affected cell types can potentially recapitulate disease pathology more accurately than existing disease models. Such successful modeling of muscular diseases requires efficient differentiation of hiPSCs into skeletal muscles. hiPSCs transduced with doxycycline-inducible *MYOD1* (*MYOD1*-hiPSCs) have been widely used; however, they require time- and labor-consuming clonal selection, and clonal variations must be overcome. Moreover, their functionality should be carefully examined. Here, we demonstrated that bulk *MYOD1*-hiPSCs established with puromycin selection rather than G418 selection showed rapid and highly efficient differentiation. Interestingly, bulk *MYOD1*-hiPSCs exhibited average differentiation properties of clonally established *MYOD1*-hiPSCs, suggesting that it is possible to minimize clonal variations. Moreover, disease-specific hiPSCs of spinal bulbar muscular atrophy (SBMA) could be efficiently differentiated via this method into skeletal muscle that showed disease phenotypes, suggesting the applicability of this method for disease analysis. Finally, three-dimensional muscle tissues were fabricated from bulk *MYOD1*-hiPSCs, which exhibited contractile force upon electrical stimulation, indicating their functionality. Thus, our bulk differentiation requires less time and labor than existing methods, efficiently generates contractible skeletal muscles, and may facilitate the generation of muscular disease models.

## Introduction

Human induced pluripotent stem cells (hiPSCs) are able to differentiate into any cell type in the body and have potential for use in clinical and research applications, such as cell-based therapies, human disease modeling, and drug discovery^1,2^. As disease-specific hiPSCs can differentiate into the affected cell types of patients, they can potentially recapitulate disease pathology more accurately than existing disease models, such as cell lines or mouse models, and are expected to overcome difficulties associated with species differences^3^. However, successful modeling of human diseases with disease-specific hiPSCs requires efficient differentiation of hiPSCs into the affected cell types. Therefore, for efficient modeling of muscular diseases, it is crucial to develop a method for rapid, efficient, and reproducible differentiation of hiPSCs into skeletal muscles.

In the last several years, a variety of protocols for skeletal muscle differentiation from human pluripotent stem cells (hPSCs), including human embryonic stem cells (hESCs) and hiPSCs, have been reported^4^. Compared with other strategies, recapitulation of skeletal muscle development in vitro with various morphogens and small molecules allows hPSCs to differentiate into skeletal muscles in a manner mimicking the process of in vivo development and induces the formation of skeletal muscles with more accurate physiological properties. Therefore, derived skeletal muscles may be applicable for mechanistic analysis of human skeletal muscle development and for regenerative therapies. However, in most cases, these differentiation protocols involve many steps that require time and labor, and the efficiency of the differentiation is rather varied^5–10^. In contrast, forced expression of transcription factors associated with skeletal muscle development, such as *PAX3* and *PAX7*, which are expressed in satellite cells, or *MYOD1*, a master gene for myogenic differentiation that induces myogenic transdifferentiation of fibroblasts^11^, more directly induces hPSCs to differentiate into skeletal muscles^12–18^. Since these transgene-based methods may not allow physiological differentiation, as they bypass important developmental processes, they may have limited applications in analyses of skeletal muscle development, modeling of muscular diseases associated with developmental abnormalities, and regenerative therapies. However, skeletal muscle differentiation via the expression of transcription factors, especially *MYOD1*, is a valuable tool for the pathophysiological analysis of muscular diseases using disease-specific hiPSCs because it is highly efficient and rapid^19^.

To date, success in skeletal muscle differentiation via *MYOD1* expression has been reported with the use of lentiviruses, adenoviruses, and *piggyBac* transposon vectors, and these methods are widely used for analyses of muscular diseases^4,14–18,20,21^. One of the tools, the *piggyBac* transposon vector for inducible expression of *MYOD1*, is transduced into hPSCs, and the cells are selected with G418 or puromycin and screened for the appropriate *MYOD1*-hPSC clones exhibiting highly efficient skeletal muscle differentiation^16,22^. This differentiation method is relatively simple but still requires a time- and labor-consuming procedure to select appropriate *MYOD1*-hPSC clones in order to achieve highly efficient differentiation, and the proportion of appropriate *MYOD1*-hPSC clones remains relatively low. Clonal variations among *MYOD1*-hPSC clones must also be considered^23^, which may result in variation in the differentiation efficiency and the variability of the phenotypes, potentially masking pathologies or drug efficacies in analyses using disease-specific hiPSCs. Therefore, multiple *MYOD1*-hPSC clones for each hPSC clone should be analyzed to confirm the reproducibility of the results. Notably, there are increasingly high expectations for analyses of sporadic diseases using disease-specific hiPSCs, such as analyses using samples from patient registries. In these cases, very large numbers of hiPSCs established from large numbers of patients and controls should be examined, but the establishment of multiple *MYOD1*-hiPSC clones for these cells is not realistic.

Based on these considerations, bulk establishment of *MYOD1*-hPSCs or bulk differentiation of muscle cells from hPSCs has been proposed to be beneficial for saving time and labor, minimizing the effects of clonal variations, and enabling quick analyses of relatively large numbers of hPSCs. Through such a protocol, unknown pathological mechanisms may be clearly visualized. However, the bulk myogenic differentiation reported thus far, achieved via transcription factor expression driven by lentiviruses, adenoviruses, or *piggyBac* transposon vectors, requires additional procedures. For example, these approaches require the induction of intermediate mesodermal progenitors and/or purification of transduced cells or muscular progenitors by flow cytometry and cell sorting (fluorescence-activated cell sorting, FACS) using surface markers or fluorescent reporters to achieve high differentiation efficiency. Without these steps, only moderate differentiation efficiency is achieved, e.g., only 40% Myogenin (MyoG)^+^ cells or 60–70% Myosin heavy chain (MHC)^+^ cells^13–15,17,18,20,24,25^. Moreover, this method cannot be used where FACS instrumentation is not available.

In this study, we demonstrated a simple and highly efficient method for contractible skeletal muscle differentiation from hiPSCs that takes advantage of bulk-established *MYOD1*-hiPSCs selected with puromycin. Puromycin selection of *MYOD1*-hiPSCs resulted in high-level transgene expression and highly efficient skeletal muscle differentiation even in bulk culture of *MYOD1*-hiPSCs, which could not be achieved by G418 selection. The relevance of bulk culture of *MYOD1*-hiPSCs was confirmed by comparing the myogenic differentiation properties of the bulk-cultured cells with those of multiple clones of *MYOD1*-hiPSCs of the same origin. Moreover, disease-specific hiPSCs of spinal bulbar muscular atrophy (SBMA) could be efficiently differentiated via this method into skeletal muscle that showed disease phenotypes, suggesting the applicability of this method for disease analysis. We also fabricated 3D muscle tissues from multiple bulk *MYOD1*-hiPSCs and found that these tissues exhibited contractile force upon electrical pulse stimulation (EPS). These results suggest that our bulk differentiation system may help to facilitate the generation of disease models for analyses of the pathophysiological mechanisms of muscular disorders.

## Results

### Establishment of clonal or bulk *MYOD1*-hiPSCs with G418 or puromycin selection

**Figure 1:**
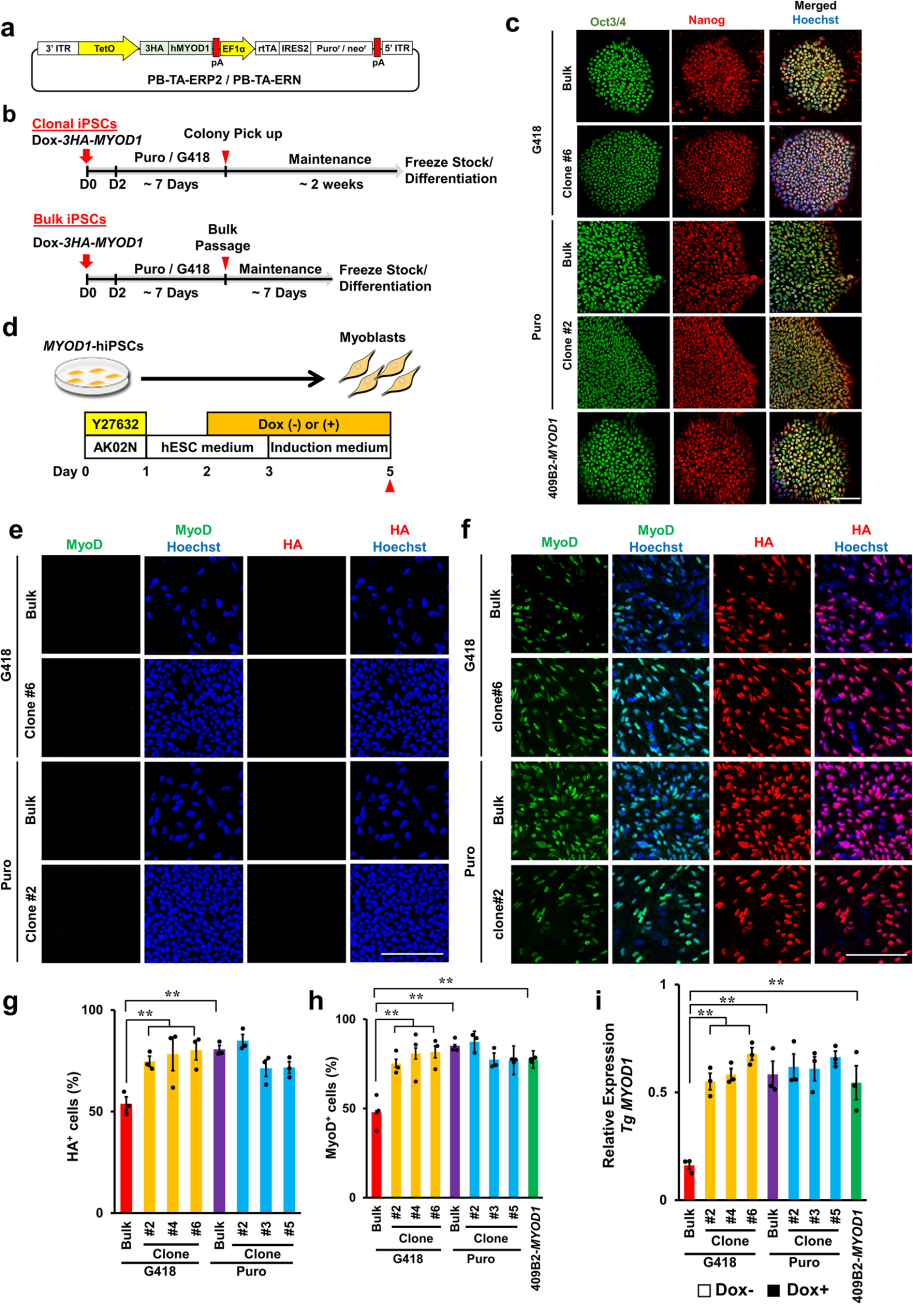
Establishment of bulk and clonal *MYOD1*-hiPSCs transduced with Dox-inducible *3HA-hMYOD1* (*Dox-3HA-MYOD1*) and Dox-inducible *3HA-hMYOD1* expression under differentiating conditions. (**a**) *PiggyBac* vector for Dox-inducible *3HA-hMYOD1* expression with the selection cassette for G418 or puromycin (*Dox-3HA-MYOD1*). (**b**) Schematic of the establishment of bulk and clonal *MYOD1*-hiPSCs. The upper panel indicates the protocol for clonal *MYOD1*-hiPSCs, and the lower panel indicates the protocol for bulk *MYOD1*-hiPSCs. (**c**) ICC analysis of the established *MYOD1*-hiPSCs for pluripotent stem cell markers (Oct3/4 and Nanog). The nuclei were stained with Hoechst 33258. All bulk and clonal *MYOD1*-hiPSCs retained the expression of pluripotent stem cell markers. Scale bar, 50 μm. (**d**) Schematic of the analysis of transgene expression under differentiation conditions with or without Dox. *MYOD1*-hiPSCs were induced to differentiate into skeletal muscles in the presence of Dox from day 2 to day 5 or cultured in the absence of Dox. Samples were collected at the times indicated by the closed triangles. (**e**–**h**) ICC analysis of the expression of transgenes (HA) and MyoD1 in cells cultured under differentiating conditions without Dox (**e**) or with Dox (**f**). Scale bar, 200 μm. Quantitative analyses of HA^+^ and MyoD1^+^ cells are shown in g and h, respectively. The expression levels of transgenes (HA) and MyoD1 were lower in the G418-bulk line than in the G418-clones but were similar in the Puro-bulk line and the Puro-clones. (**i**) Transgene expression in bulk and clonal *MYOD1*-hiPSCs established with G418 or puromycin selection in the differentiating conditions in the presence of Dox as examined by qRT‒PCR and compared with that of control 409B2-*MYOD1*-hiPSCs. The amount of cDNA was normalized to that of human-specific *β-ACTIN*. The G418-bulk line expressed transgenes at only approximately 25% of the level observed in the G418 clones or 409B2-*MYOD1*-hiPSCs. The data are presented as the mean ± SEM, n = 3. *p < 0.05, **p < 0.01. ANOVA followed by post hoc Bonferroni test.

To investigate whether we could efficiently derive skeletal muscles from bulk culture of *MYOD1*-hiPSCs without clonal selection and to determine how different selection markers, namely, G418 and puromycin, affect the myogenic differentiation efficiency of *MYOD1*-hiPSCs, both bulk and clonal *MYOD1*-hiPSCs were established through transduction of a *piggyBac* transposon vector for the doxycycline (Dox)-inducible expression of human *MYOD1* tagged to *3*× *HA* (*Dox-3HA-hMYOD1*) (with a selection cassette for G418 or puromycin) into hiPSCs (201B7)^2^ (PB-TA-*3HA-hMYOD1*-ERN or PB-TA-*3HA-hMYOD1*-ERP2) (Fig. 1a,b). Ultimately, one bulk *MYOD1*-hiPSC line and six *MYOD1*-hiPSC clones were established each by G418 selection and puromycin selection (resulting in a G418-bulk line, a Puro-bulk line, G418-clones, and Puro-clones). All established bulk and clonal *MYOD1*-hiPSCs were morphologically well maintained in the undifferentiated state and exhibited characteristic hESC-like morphology. We also confirmed that they expressed the hPSC markers Oct3/4 and Nanog by immunocytochemical (ICC) analysis, suggesting that all the bulk and clonal *MYOD1*-hiPSCs retained pluripotency (Fig. 1c and Supplementary Fig. S1).

### Dox-inducible expression of *3HA-hMYOD1* transgenes in undifferentiated and differentiating conditions

To confirm Dox-inducible expression of the *3HA-hMYOD1* transgenes, 1.5 µg/ml Dox was added 2 days after the passage of all the *MYOD1*-hiPSC clones and bulk lines, and the cells were maintained under undifferentiated conditions for 3 days. Next, the expression of transgenes in the cells was analyzed in the presence and absence of Dox (Supplementary Fig. S2a). In the absence of Dox, the expression of the transgenes (HA) and MyoD1 could not be detected in any of the *MYOD1*-hiPSC clones or bulk lines by ICC analysis (Supplementary Fig. S2b). In the presence of Dox, the proportions of HA^+^ and MyoD1^+^ cells in the G418-bulk line were 47.3 ± 5.3% and 46.5 ± 5.2%, respectively, whereas those in the G418-clones were significantly higher at approximately 65% (64.1 ± 2.2 to 67.6 ± 1.7%) and 75% (74.3 ± 1.1 to 79.5 ± 0.5%), respectively (Supplementary Fig. S2c–e). In contrast, the proportions of HA^+^ and MyoD1^+^ cells in the Puro-bulk line were 82.2 ± 1.4% and 80.0 ± 2.5%, respectively, and were not significantly different from those in the Puro-clones, in which the proportions of HA^+^ and MyoD1^+^ cells were both approximately 80% (75.1 ± 1.0 to 80.3 ± 1.9% and 76.8 ± 2.4 to 83.2 ± 2.9%, respectively) (Supplementary Fig. S2c–e). Dox-inducible expression of the transgene (*3HA-hMYOD1*) was also examined by quantitative RT‒PCR (qRT‒PCR) in comparison with that in control 409B2-*MYOD1*-hiPSCs (409B2*-MYOD1*) that had been clonally established with PB-TAG-*hMYOD1*-ERP (puromycin selection, without the 3× HA tag)^22^ and were shown to differentiate into skeletal muscles with high efficiency. Consistent with the results of the ICC analysis, in the presence of Dox, the expression of transgenes in the G418-bulk line was significantly lower than that in the G418-clones and 409B2-*MYOD1*-hiPSCs (approximately 50% of the G418-clones and 409B2-*MYOD1*-hiPSCs) (Supplementary Fig. S2f). In contrast, the Puro-bulk line expressed the same levels of transgenes as Puro-clones and 409B2-*MYOD1*-hiPSCs, and the transgene expression levels in these *MYOD1*-hiPSCs in the presence of Dox were significantly higher than those in the G418-bulk line (Supplementary Fig. S2f). Taken together, these results suggest that in undifferentiated states, the G418-bulk line was not capable of expressing sufficient levels of transgenes in response to Dox, unlike the G418-clones and 409B2-*MYOD1*-hiPSCs. In contrast, Puro-bulk *MYOD1*-hiPSCs were able to express sufficient amounts of transgenes in response to Dox that were comparable to the levels in the Puro-clones and 409B2-*MYOD1*-hiPSCs. To investigate the copy numbers of integrated transgenes in each bulk and clonal *MYOD1*-hiPSCs, genomic PCR was performed using the primers for *MYOD1*-transgenes. There did not appear to be obvious correlations between the copy numbers of integrated transgenes and the expression levels of the transgenes (Supplementary Fig. S2g).

Considering the silencing of transgenes during differentiation, Dox-inducible transgene expression was also evaluated in differentiating hiPSCs (Fig. 1d). *MYOD1*-hiPSCs were differentiated into skeletal muscles as reported previously with modifications^16^. From day 2 of differentiation, *MYOD1*-hiPSCs were cultured in the presence or absence of 1.5 μg/ml Dox for 3 days to induce the expression of *3HA-hMYOD1* (Fig. 1d). The transgene and endogenous *hMYOD1* expression was then analyzed by ICC analysis and qRT‒PCR. In the absence of Dox, the expression of transgenes (HA) and MyoD1 was not detected in any of the *MYOD1*-hiPSC bulk lines or clones (Fig. 1e). Consistent with the results obtained in the undifferentiated condition, in response to Dox, the cells derived from the G418-bulk line were composed of only approximately 50% HA^+^ or MyoD1^+^ cells (53.9 ± 3.4% for HA, 48.0 ± 6.0% for MyoD1), whereas those derived from the G418-clones were composed of more than 75% HA^+^ or MyoD1^+^ cells (74.2 ± 2.5% to 80.2 ± 4.8% for HA, 75.3 ± 3.7% to 81.7 ± 4.9% for MyoD1), which was comparable to the results obtained with 409B2-*MYOD1*-hiPSCs for MyoD1 expression (77.5 ± 0.7%) (Fig. 1f–h). In contrast, the Puro-bulk line produced HA^+^ and MyoD1^+^ cells (80.6 ± 1.9% and 85.2 ± 2.3%, respectively) to the same extent as the Puro-clones, which yielded approximately 80% HA^+^ and MyoD1^+^ cells (71.4 ± 4.1% to 84.9 ± 3.0% for HA, 76.9 ± 0.6% to 87.3 ± 3.1% for MyoD1). This result was relatively similar to the MyoD1 result for the 409B2-*MYOD1*-hiPSCs (Fig. 1f–h). Similar results were obtained by qRT‒PCR analysis. In response to Dox, the G418-bulk line expressed transgenes at only approximately 25% of the levels in G418-clones or 409B2-*MYOD1*-hiPSCs, in contrast to the Puro-bulk line, which expressed transgenes at levels comparable to those in the Puro-clones and 409B2-*MYOD1*-hiPSCs (Fig. 1i). Thus, in the differentiating condition, the G418-bulk line did not express *3HA-hMYOD1* transgenes sufficiently in response to Dox, whereas the Puro-bulk line, as well as the G418-clones and the Puro-clones, was capable of expressing higher levels of transgenes. Because sufficient expression of transgenes is required for efficient differentiation into skeletal muscles in this system, these results suggest that the selection of appropriate *MYOD1*-hiPSC clones is essential when *MYOD1*-hiPSCs are established with G418 selection, whereas clonal selection may not be required when the *MYOD1*-hiPSCs are established with puromycin selection.

### Bulk *MYOD1*-hiPSCs established with puromycin selection were able to efficiently differentiate into mature skeletal muscles as clonally established *MYOD1*-hiPSCs

**Figure 2:**
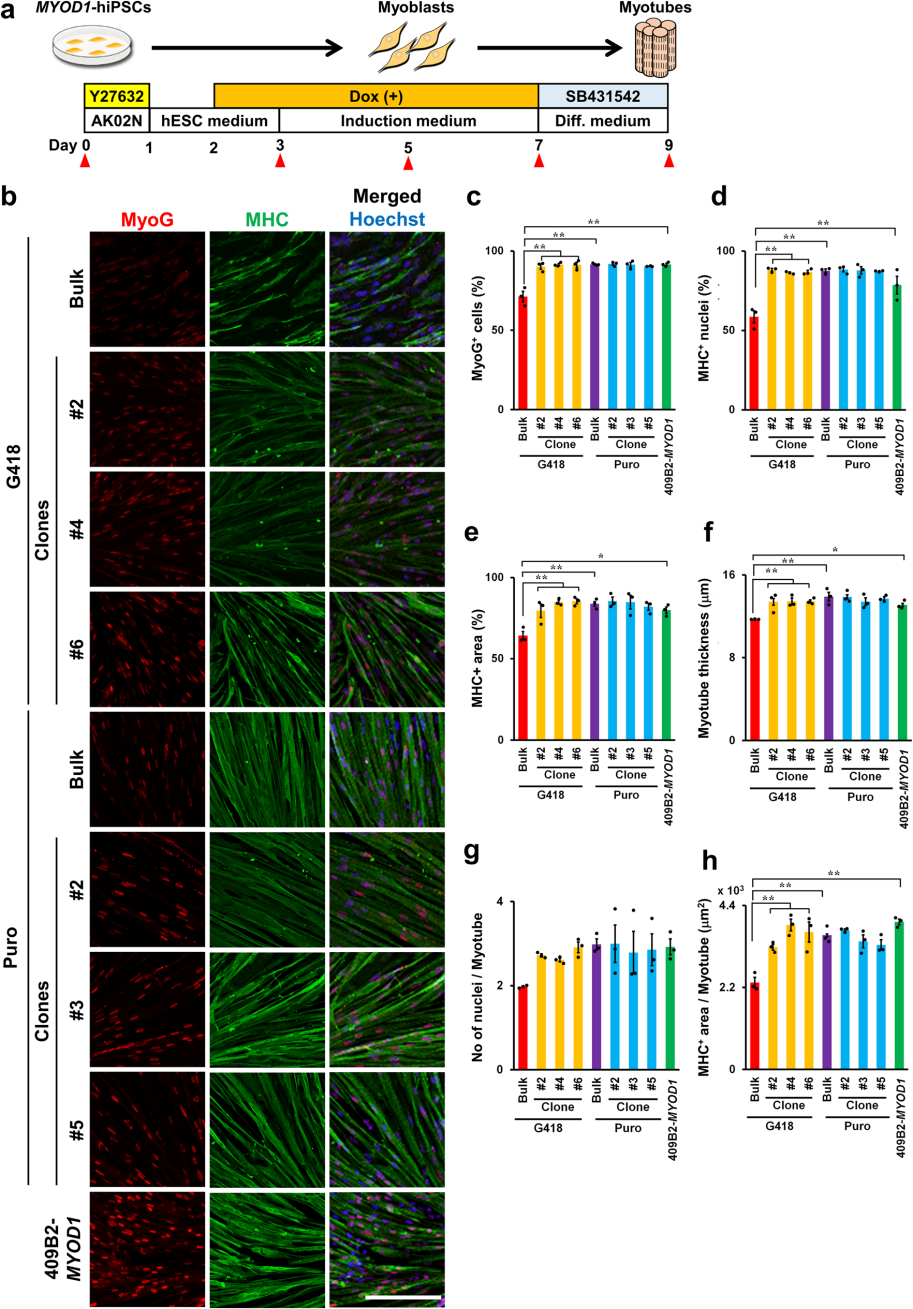
Bulk *MYOD1*-hiPSCs established with puromycin selection achieved efficient myogenic differentiation and myotube maturation similar to that achieved by clonally established *MYOD1*-hiPSCs. (**a**) Schematic presentation of skeletal muscle differentiation from *MYOD1*-hiPSCs. Samples were collected at the times indicated with the closed triangles. (**b**) ICC analysis of myotubes derived from bulk and clonal *MYOD1*-hiPSCs established with G418 or puromycin selection and control 409B2-*MYOD1*-hiPSCs for the expression of MyoG and MHC at day 9 of differentiation. The nuclei were stained with Hoechst 33258. Scale bar, 200 μm. (**c**–**e**) Quantitative analysis of the parameters for skeletal muscle differentiation, including the proportions of MyoG^+^ cells among total cells (**c**), the proportions of MHC^+^ nuclei among total nuclei (**d**), and the proportion of MHC^+^ area in the total area (**e**). Puro-bulk *MYOD1*-hiPSCs exhibited differentiation potential similar to that of clonal *MYOD1*-hiPSCs and control 409B2-*MYOD1*-hiPSCs, whereas G418-bulk *MYOD1*-hiPSCs exhibited lower differentiation potential than the other *MYOD1*-hiPSCs. (**f**–**h**) Quantitative analysis of the parameters for myotube maturation, including the myotube thickness (**f**), number of nuclei per myotube (**g**), and MHC^+^ area per myotube (**h**). Puro-bulk *MYOD1*-hiPSCs exhibited myotube maturation potential similar to that of clonal *MYOD1*-hiPSCs and control 409B2-*MYOD1*-hiPSCs, whereas G418-bulk *MYOD1*-hiPSCs exhibited lower myotube maturation potential than the other *MYOD1*-hiPSCs. The data are presented as the mean ± SEM, n = 3. *p < 0.05, **p < 0.01. ANOVA followed by post hoc Bonferroni test.

To confirm the correlation between transgene expression and the potential to differentiate into skeletal muscles, all bulk *MYOD1*-hiPSCs and clonal *MYOD1*-hiPSCs were differentiated into myotubes to evaluate differentiation potential and myotube maturation (Fig. 2a). As expected, 2 days after Dox withdrawal (day 7), ICC analysis revealed that the G418-bulk line, which failed to sufficiently express *3HA-hMYOD1* transgenes in response to Dox, showed inefficient differentiation into myotubes, as indicated by the significantly lower proportions of MyoG^+^ cells (71.2 ± 3.3%), MHC^+^ nuclei (58.6 ± 3.8%), and MHC^+^ area (64.2 ± 2.6%) than in the G418-clones (MyoG^+^ cells: 90.2 ± 1.7% to 91.6 ± 0.8%, MHC^+^ nuclei: 78.6 ± 5.5% to 88.2 ± 0.9%, MHC^+^ area: 79.5 ± 4.2% to 85.4 ± 1.5%) and control 409B2-*MYOD1*-hiPSCs (MyoG^+^ cells: 91.6 ± 0.8%, MHC^+^ nuclei: 78.6 ± 5.5%, MHC^+^ area: 80.0 ± 2.0%) (Fig. 2b–e, Supplementary Fig. S3a,b). On the other hand, the Puro-bulk line exhibited 91.6 ± 0.4% MyoG^+^ cells, 87.6 ± 1.1% MHC^+^ nuclei, and 83.8 ± 1.4% MHC^+^ area after differentiation, which were comparable to those of the Puro-clones (MyoG^+^ cells: 90.4 ± 0.2% to 91.8 ± 0.8%, MHC^+^ nuclei: 87.3 ± 0.3% to 88.4 ± 1.4%, MHC^+^ area: 82.0 ± 2.2% to 85.5 ± 2.4%) and control 409B2-*MYOD1*-hiPSCs (Fig. 2b–e, Supplementary Fig. S3a,b). These results suggest that Puro-bulk *MYOD1*-hiPSCs, which expressed transgenes at sufficient levels, were able to differentiate into skeletal muscles as efficiently as clonally established *MYOD1*-hiPSCs, but G418-bulk *MYOD1*-hiPSCs were not able to do so because they lacked sufficient transgene expression. Moreover, quantitative analysis of the parameters of mature myotubes, including the myotube thickness, the number of nuclei per myotube, and the MHC^+^ area per myotube, revealed that the G418-bulk line exhibited significantly poorer maturation according to all the parameters (myotube thickness: 11.7 ± 0.1 μm, MHC^+^ nuclei per myotube: 2.0 ± 0.02, MHC^+^ area per myotube: 2333.3 ± 137.2 μm^2^) than G418-clones or control 409B2-*MYOD1*-hiPSCs (myotube thickness: 13.4 ± 0.3 μm to 13.5 ± 0.1 μm, MHC^+^ nuclei per myotube: 2.6 ± 0.1 to 2.9 ± 0.1, MHC^+^ area per myotube: 3283.1 ± 77.3 μm^2^ to 3960.0 ± 75.8 μm^2^). In contrast, the Puro-bulk line gave rise to mature skeletal muscles (myotube thickness: 13.9 ± 0.4 μm, MHC^+^ nuclei per myotube: 3.0 ± 0.1, MHC^+^ area per myotube: 3604.8 ± 35 μm^2^ MHC^+^) as efficiently as the Puro-clones (myotube thickness: 13.9 ± 0.2 μm to 13.4 ± 0.4 μm, MHC^+^ nuclei per myotube: 2.8 ± 0.5 to 3.0 ± 0.4, MHC^+^ area per myotube: 3440.7 ± 173.2 μm^2^ to 3742.5 ± 31.8 μm^2^) and 409B2-*MYOD1*-hiPSCs (Fig. 2b,f–h, Supplementary Fig. S3a,b). These results suggest that puromycin selection for the establishment of *MYOD1*-hiPSCs facilitates higher transgene expression than G418 selection, which is critically correlated with the efficiency of myogenic differentiation of *MYOD1*-hiPSCs as well as maturation of iPSC-derived myotubes.

### Bulk *MYOD1*-hiPSCs exhibited an average differentiation process compared to that of clonal *MYOD1*-hiPSCs and may have minimized the effects of clonal variations

**Figure 3:**
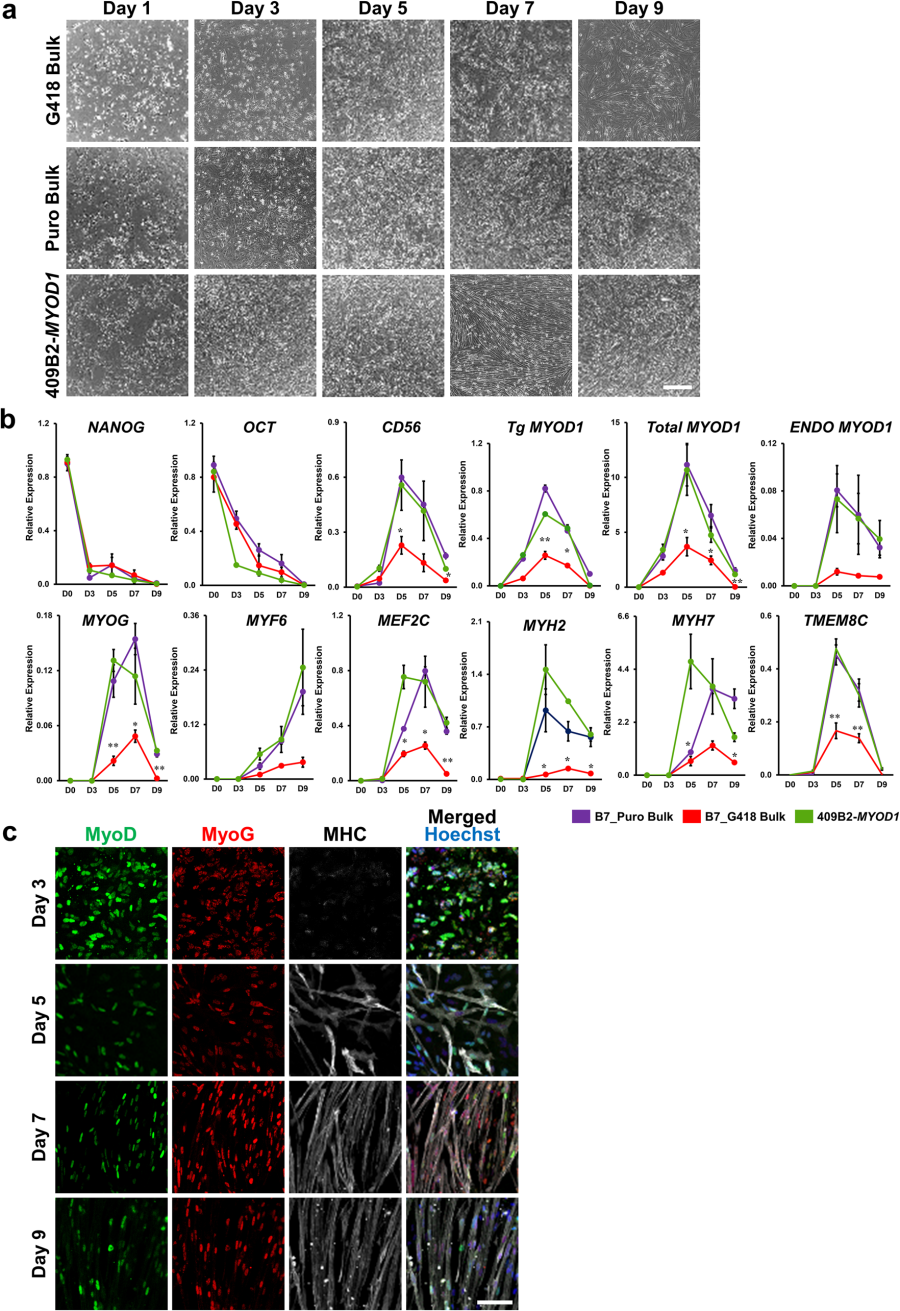
Time course analysis revealed highly efficient myogenic differentiation of Puro-bulk *MYOD1*-hiPSCs. (**a**) Brightfield images showing the time course of myogenic differentiation of G418-bulk and Puro-bulk *MYOD1*-hiPSCs as well as control 409B2-*MYOD1*-hiPSCs. After day 7, G418-bulk *MYOD1*-hiPSCs featured nonmuscle cells with flattened morphology and showed fewer myotubes and larger spaces among myotubes than Puro-bulk and clonal *MYOD1*-hiPSCs. Scale bar, 100 μm. (**b**) Time course gene expression analysis of G418-bulk, Puro-bulk, and control 409B2-*MYOD1*-hiPSCs along with myogenic differentiation. The amount of cDNA was normalized to that of human-specific *β-ACTIN* and is presented as the expression relative to that in undifferentiated hiPSCs (*NANOG* and *OCT3/4*), that in the human myoblast cell line Hu5/KD3 differentiated for 3 days (*CD56*, total and endogenous *MYOD1*, *MYOG*, *MYF6*, *MEF2C*, *MYH2*, *MYH7*, and *TMEM8C*), or that in EKN3-*MYOD1*-hiPSCs differentiated for 5 days (*Tg MYOD1*). The data are presented as the mean ± SEM, n = 3. *, *p* < 0.05, **, *p* < 0.01 vs*.* 409B2-*MYOD1*-hiPSCs. ANOVA followed by post hoc Bonferroni test. (**c**) Time course ICC analysis of Puro-bulk *MYOD1*-hiPSCs for MyoD, MyoG, and MHC along with myogenic differentiation. The number of MyoD^+^ cells gradually decreased after day 7, while MHC^+^ cells appeared around day 5 and formed aligned myotubes from day 7. Scale bar, 100 μm.

To examine the differences between bulk and clonal *MYOD1*-hiPSCs and G418 and puromycin selection in more detail, we further performed time-course analysis of skeletal muscle differentiation with all *MYOD1*-hiPSC bulk lines and clones. As differentiation progressed, all the bulk and clonal *MYOD1*-hiPSCs gradually showed morphological differentiation into myoblasts and subsequently into myotubes, and most of the cells formed myotube-like aligned structures by day 7, the time of Dox withdrawal. As expected, clonal *MYOD1*-hiPSCs and Puro-bulk *MYOD1*-hiPSCs exhibited further maturation of myotubes, whereas G418-bulk *MYOD1*-hiPSCs showed increased spaces among myotubes and decreased numbers of myotubes and gave rise to nonmuscle cells with a more flattened morphology (Fig. 3a, Supplementary Fig. S3c,d). Time-course gene expression analysis of markers associated with skeletal muscle development was also performed in the presence of Dox by qRT‒PCR (Fig. 3b and Supplementary Fig. S4). The expression of pluripotent stem cell markers, including *NANOG* and *OCT3/4*, gradually and similarly decreased in all *MYOD1*-hiPSCs over the course of differentiation regardless of the method used for *MYOD1*-hiPSC establishment. In all the bulk and clonal *MYOD1*-hiPSCs, the expression of the premyogenic marker CD56 and the core myogenic regulatory factor (MRF) total *MYOD1*, including endogenous *MYOD1* and transgenes, appeared on day 3, just after the induction of the transgenes, and gradually increased to peak on day 5, after which it decreased. Endogenous *MYOD1* expression peaked at day 5, just after transgene induction, and was downregulated thereafter; however, it was maintained at approximately 50% of the peak expression even after the withdrawal of Dox at day 7. Other MRFs, including *MYOG* and *MYF6*, which are crucial for the fate determination and terminal differentiation of skeletal muscles ^26,27^, showed gradual increases in their expression along with differentiation. *MYOG* showed decreased expression after the withdrawal of Dox at day 7, whereas *MYF6* showed continuous upregulation thereafter. The expression of *MYF5* was not detected at all, consistent with previous reports^14–16^, probably due to negative feedback regulation from the overexpression of *MYOD1,* which has similar roles in myogenic differentiation. With regard to the maturation of myotubes, a transcription factor critically involved in the maintenance of skeletal muscles, *MEF2C*; the mature skeletal muscle markers *MYH2* and *MYH7*; and the fusion marker *TMEM8C* showed gradual increases, with peak expression from day 5 to day 7, followed by decreases in their expression thereafter. The expression of mature skeletal muscle markers, except for *TMEM8C*, was maintained at approximately 50% of the peak expression even after the withdrawal of Dox at day 7, similar to the pattern for endogenous *MYOD1*. This finding suggests that the expression of these molecules depends on *MYOD1* expression and may contribute to the continuous maturation of the cells at later stages of differentiation.

When bulk and clonal *MYOD1*-hiPSCs with G418 and puromycin selection were compared, the Puro-bulk line showed expression similar to that of the Puro-clones and 409B2-*MYOD1*-hiPSCs, whereas the G418-bulk line showed lower expression levels than other *MYOD1*-hiPSCs throughout the differentiation process. These findings are consistent with the data showing the poor differentiation potential of the G418-bulk line. More importantly, in contrast to the large clonal variations observed in differentiating Puro-clones, the Puro-bulk line showed average expression of genes associated with skeletal muscle differentiation among the six Puro clones or similar expression to that in six G418-clones during the differentiation processes (Supplementary Fig. S4). Considering the average transgene expression in undifferentiated and differentiating conditions and the average values of the parameters of skeletal muscle differentiation and maturation in the Puro-bulk line among Puro-clones and G418-clones (Figs. 1g–i, 2b–h, Supplementary Fig. S2d–f), these results suggest that Puro-bulk *MYOD1*-hiPSCs have average properties of clonal *MYOD1*-hiPSCs. This averaging may minimize the effects of clonal variation among *MYOD1*-hiPSC clones, which is responsible for a bottleneck in analyses of disease-specific hiPSCs.

Finally, we performed time-course ICC analysis of the differentiation of Puro-bulk *MYOD1*-hiPSCs to determine the differentiation potential of the cells (Fig. 3c). We found that the expression of MyoG was first observed at day 3 and was followed by the expression of MHC at day 5. Morphological alteration into aligned myotubes was observed from day 7 of differentiation, which was consistent with the results of time-course morphological and qRT‒PCR analyses (Fig. 3a,b). Taken together, these results suggested that bulk *MYOD1*-hiPSCs established by puromycin selection were capable of differentiating into mature skeletal muscles as efficiently as clonally established *MYOD1*-hiPSCs.

### Reproducibility of more efficient myogenic differentiation in Puro-bulk *MYOD1*-hiPSCs than G418-bulk *MYOD1*-hiPSCs

**Figure 4:**
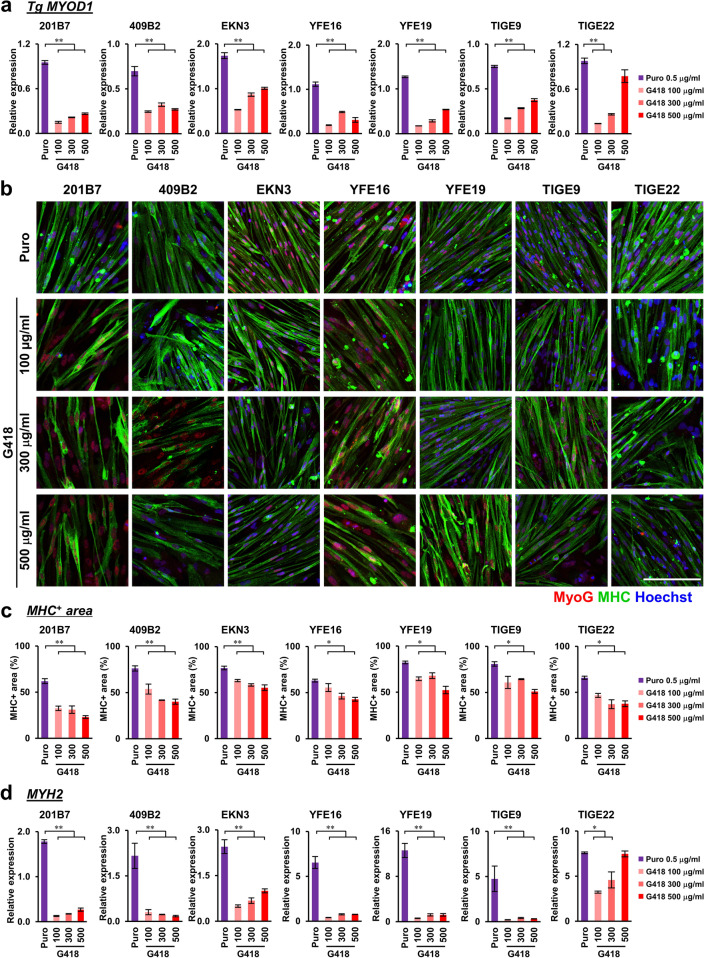
Reproducibility of higher transgene expression and more efficient skeletal muscle differentiation in seven bulk *MYOD1*-hiPSCs established by puromycin selection compared with those established by selection with various concentrations of G418. (**a**) Transgene expression in seven *MYOD1*-hiPSC clones (201B7, 409B2, EKN3, YFE16, YFE19, TIGE9, and TIGE22) established with puromycin (0.5 μg/ml) or G418 (100 μg/ml, 300 μg/ml, 500 μg/ml) selection in the differentiating conditions (day 5) in the presence of Dox as examined by qRT‒PCR. The amount of cDNA was normalized to that of human-specific *β-ACTIN*. The G418-bulk line exhibited poor transgene expression even when the cells were selected with high concentrations of G418. The data are presented as the mean ± SEM, n = 3. *, *p* < 0.05, **, *p* < 0.01. ANOVA followed by post hoc Bonferroni test. (**b**) ICC analysis of myotubes derived from bulk-*MYOD1*-hiPSCs established from seven hiPSC clones by selection with puromycin or various concentrations of G418 for the expression of MyoG and MHC at day 9 of differentiation. The nuclei were stained with Hoechst 33258. All seven Puro-bulk *MYOD1*-hiPSC lines efficiently differentiated into skeletal muscles in the presence of Dox, whereas G418-bulk *MYOD1*-hiPSC lines showed moderate or poor differentiation potential regardless of the concentration of G418 used for the selection. Scale bar, 200 μm. (**c**) Quantitative analysis of the parameter for skeletal muscle differentiation, the proportion of MHC^+^ area in the total area. The data are presented as the mean ± SEM, n = 3. *, *p* < 0.05, **, *p* < 0.01. ANOVA followed by post hoc Bonferroni test. (**d**) Expression of *MYH2* in bulk *MYOD1*-hiPSC lines established from seven hiPSC clones by selection with puromycin (0.5 μg/ml) or various concentrations of G418 (100 μg/ml, 300 μg/ml, 500 μg/ml) at day 9 of differentiation. The amount of cDNA was normalized to that of human-specific *β-ACTIN* and is presented as the relative expression in the human myoblast cell line Hu5/KD3 differentiated for 3 days. The data are presented as the mean ± SEM, n = 3. *, *p* < 0.05, **, *p* < 0.01. ANOVA followed by post hoc Bonferroni test.

To confirm the higher transgene expression and more efficient differentiation of bulk *MYOD1*-hiPSCs generated via puromycin selection in comparison with those generated via G418 selection and to examine the effects of higher concentrations of G418, seven hiPSC clones (201B7, 409B2, EKN3, YFE16, YFE19, TIGE9, and TIGE22) established from fibroblasts of adult healthy individuals^28–30^ were transduced with the *Dox-3HA-hMYOD1 piggyBac* vector with a puromycin or a G418 selection cassette and selected for puromycin (0.5 μg/ml) or various concentrations of G418 (100, 300, or 500 μg/ml), respectively, to establish *MYOD1*-hiPSCs. Then, *MYOD1*-hiPSCs were differentiated into skeletal muscles in a bulk manner following the same protocol (Figs. 1d and 2a). ICC analysis at day 5 of differentiation revealed that all the Puro-bulk *MYOD1*-hiPSCs (Puro-bulk lines) gave rise to significantly higher proportions of HA^+^ and MyoD1^+^ cells than G418-bulk *MYOD1*-hiPSCs (G418-bulk lines) (more than 85% in Puro-bulk lines vs. less than 65% in G418-bulk lines, n = 3, *p* < 0.01) (Supplementary Fig. S5a–c). Similarly, qRT‒PCR analysis revealed that the Puro-bulk lines expressed significantly higher levels of transgene (*3HA-hMYOD1*) than the G418-bulk lines, which expressed less than half the levels in the Puro-bulk lines (n = 3, *p* < *0.01*); however, TIGE22 established with the highest concentration of G418 (500 μg/ml) expressed relatively high levels of transgene (approximately 80% of that of Puro-bulk) (Fig. 4a). Some hiPSC lines showed a G418 concentration-dependent increase in the number of cells expressing the transgene and the transgene expression level, while some hiPSC lines did not show such a correlation (Fig. 4a, Supplementary Fig. S5a–c). With respect to the integration of transgenes into the genome, some of the *MYOD1*-hiPSCs established by puromycin selection (409B2, YFE16, TIGE9) exhibited higher copy numbers of integrated transgenes than those established by G418 selection, but the copy numbers did not show clear correlations with the transgene expression levels (Fig. 4a, Supplementary Fig. S5d). Some of the *MYOD1*-hiPSCs established with G418 selection (EKN3, YFE16, TIGE9) exhibited G418 concentration-dependent increases in the copy numbers of integrated transgenes, which appeared to correlate with the expression levels of transgenes. In other hiPSCs, however, there appeared to be no apparent correlations between the copy numbers of transgenes and the transgene expression levels (Fig. 4a, Supplementary Fig. S5d). Thus, the copy numbers of integrated transgenes do not necessarily correlate with transgene expression and differentiation efficiency.

Moreover, ICC analysis at day 9 of differentiation revealed that all the Puro-bulk lines showed highly efficient differentiation into skeletal muscles, as confirmed by the proportions of MyoG^+^ cells, MHC^+^ nuclei, and MHC^+^ areas of more than 90%, 85%, and 80%, respectively; in contrast, G418-bulk lines exhibited less efficient differentiation potential, as shown by the same parameters of less than 80%, 75% and 70%, respectively, even with high concentrations of G418 (Fig. 4b,c, Supplementary Fig. S6a,b). Unexpectedly, TIGE22-*MYOD1*-hiPSCs established with a high concentration of G418 (500 μg/ml) did not show a differentiation potential as high as those established with puromycin, especially with regard to parameters such as MHC^+^ area, despite their high transgene expression (Fig. 4a–c, Supplementary Figs. S5a–c, S6a,b). Similar results were obtained by qRT‒PCR analysis at day 9 of differentiation, in which cells derived from most of the Puro-bulk lines, except for TIGE22 and EKN3, exhibited increased expression of myogenic genes such as *MYF6, MYOG*, *MYH2*, and *MYH7* compared to those derived from the corresponding G418-bulk lines (in most cases more than twofold, in some cases more than tenfold) (Fig. 4d, Supplementary Fig. S6c–e). Cells derived from G418-bulk lines established from TIGE22 (300, 500 μg/ml) and EKN3 (500 μg/ml) with higher concentrations of G418 expressed *MYH7* and *MYH2* or *MYH7*, respectively, as highly as cells derived from the corresponding Puro-bulk lines (Fig. 4d, Supplementary Fig. S6c). However, the expression levels of other genes and the ICC results suggested that the skeletal muscle differentiation potential of the G418-bulk lines, even in these lines, was lower than that of the Puro-bulk lines (Fig. 4b–d, Supplementary Fig. S6).

Further analysis was performed to quantify the myotube thickness, number of nuclei per myotube, and MHC^+^ area per myotube and revealed that all the Puro-bulk lines exhibited a greater potential to differentiate into mature myotubes than the G418-bulk lines, which showed poorer maturation (Supplementary Fig. S7a–c). Finally, by ICC analysis of α-actinin at day 9 of differentiation, sarcomere formation was clearly visualized in myotubes derived from all Puro-bulk *MYOD1*-hiPSCs, suggesting that these hiPSC-derived myotubes were mature enough to exhibit functional properties (Supplementary Fig. S7d)^31^. These results suggest that the highly efficient myogenic differentiation and maturation of Puro-bulk *MYOD1*-hiPSCs in contrast to G418-bulk *MYOD1*-hiPSCs is reproducible and applicable to various hiPSCs for simple and efficient mature skeletal muscle differentiation.

### Transcriptome analysis revealed more efficient differentiation of Puro-Bulk *MYOD1*-hiPSCs into mature skeletal muscles

**Figure 5:**
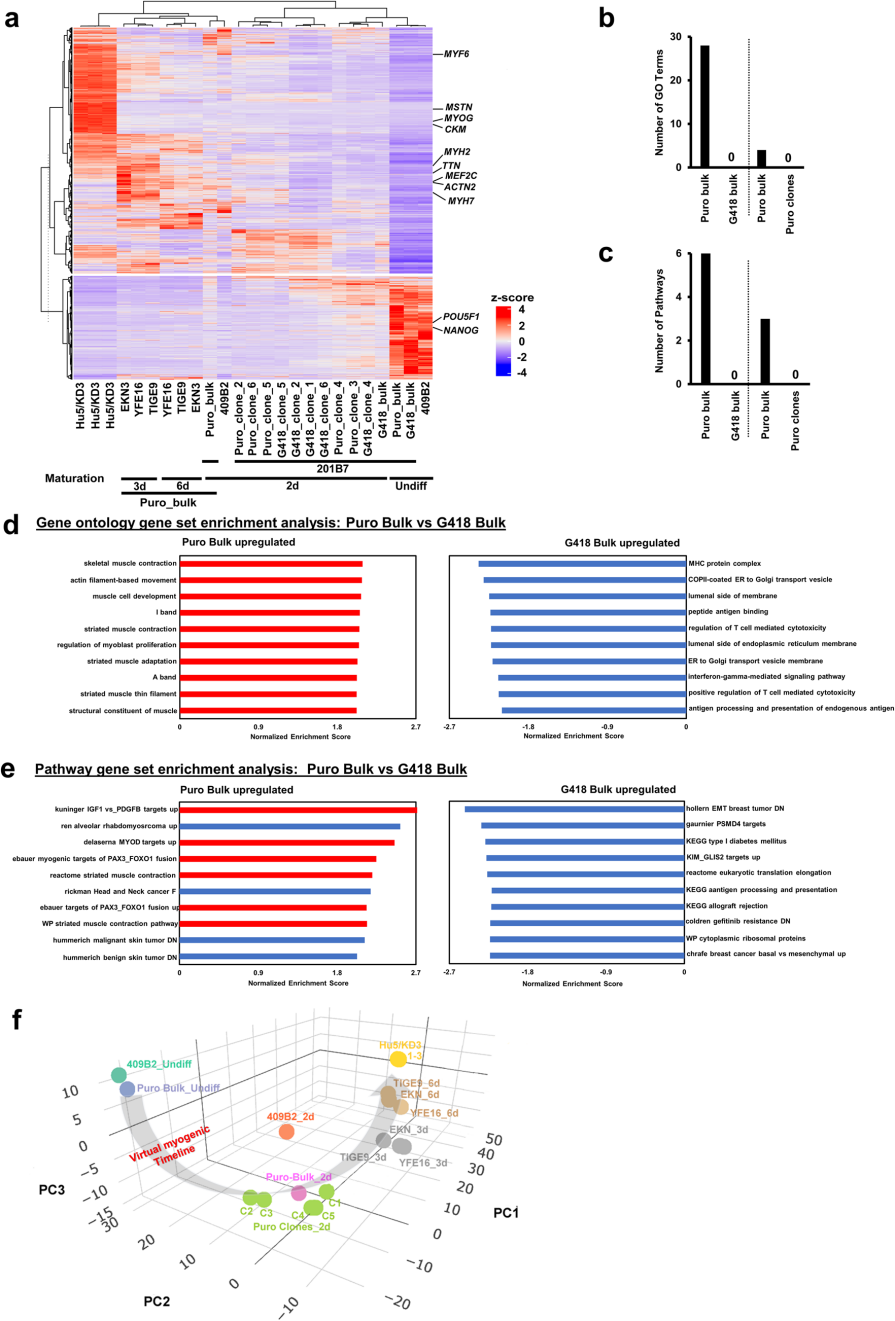
Transcriptome analyses revealed that Puro-bulk *MYOD1*-hiPSCs were capable of differentiating into more mature skeletal muscles. (**a**) Hierarchical clustering based on 2,794 genes identified as having significantly altered expression between undifferentiated *MYOD1*-hiPSCs (day 0) and skeletal muscles derived from control hiPSCs (409B2-*MYOD1*) (day 9) or human myoblast cell lines (Hu5/KD3). 2d, 3d, and 6d: 2 days, 3 days, and 6 days in myotube differentiation medium, respectively; Undiff.: undifferentiated iPSCs. The expression data were grouped using a hierarchical clustering algorithm (ward. D2) by average linkage with the Pearson distance and visualized by ComplexHeatmap ver. 2.13.1 (https://github.com/jokergoo/ComplexHeatmap)^65^ and dendsort ver. 0.3.4 (https://github.com/evanbiederstedt/dendsort)^66^. (**b**) Quantitative analysis of the number of skeletal muscle-related GO terms significantly enriched in each sample indicated (Puro-bulk vs. G418-bulk and Puro-bulk vs. Puro-clones) (FDR *q*-value < 0.01). (**c**) Quantitative analysis of the number of skeletal muscle-related pathways significantly enriched in each sample indicated (Puro-bulk vs. G418-bulk and Puro-bulk vs. Puro-clones) (FDR* q*-value < 0.01). (**d**) GO enrichment analysis. The top 10 upregulated gene sets in Puro- and G418-bulk with *FDR q*-values < 0.01 are shown. The red bar indicates muscle-related gene sets, and the blue bar indicates non-muscle-related gene sets. (**e**) Pathway gene set enrichment analysis. The top 10 pathways significantly enriched in Puro- and G418-bulk with *FDR q*-values < 0.01 are shown. The red bar indicates muscle-related pathways, and the blue bar indicates non-muscle-related pathways. (**f**) 3D image of the PCA based on 10,000 genes among undifferentiated *MYOD1*-hiPSCs (409B2_Undiff, Puro-bulk_Undiff), skeletal muscles derived from control iPSCs (409B2-*MYOD1*), 201B7-*MYOD1*-hiPSCs (Puro-bulk and 5 Puro-clones) (2 days in myotube differentiation medium), various Puro-bulk *MYOD1*-hiPSCs (TIGE9-, EKN3-, and YFE16) (3 and 6 days in myotube differentiation medium), and human myoblast cell line (Hu5/KD3)-derived skeletal muscles. The distinct distributions of undifferentiated cells and differentiated skeletal muscles are indicated. The gray arrow indicates the ‘virtual myogenic timeline’. 2d, 3d, and 6d: 2 days, 3 days, and 6 days in myotube differentiation medium, respectively; Undiff. : undifferentiated iPSCs; C1-C6: clones 1 to 6 of Puro-clones.

To better understand the potential of Puro-bulk *MYOD1*-hiPSCs and comprehensively assess the distinct gene expression profiles of skeletal muscles derived from *MYOD1*-hiPSCs established in a different way, we performed global transcriptome analysis by RNA sequencing (RNA-seq) (Fig. 5). A total of 2794 genes were identified as having significantly altered expression between undifferentiated *MYOD1*-hiPSCs (Day 0) and control iPSC (409B2-*MYOD1*)-derived skeletal muscles (Day 9) or human myoblast cell line (Hu5/KD3)-derived muscle cells, which were used for hierarchical clustering (see “Materials and methods” for details; the genes are listed in Supplementary Table S4). As expected, Puro-bulk *MYOD1*-hiPSC-derived skeletal muscles exhibited expression profiles more similar to those of control 409B2-*MYOD1*-hiPSC- and Hu5/KD3-derived skeletal muscles than to those derived from Puro-clones, G418-clones, and G418-bulk-*MYOD1*-hiPSCs (Fig. 5a). Notably, G418-bulk, which exhibited poor differentiation potential into skeletal muscles, and some G418/Puro-clones still expressed a group of genes that are highly expressed in undifferentiated hiPSCs even after differentiation, suggesting that they may not have sufficiently differentiated. The remaining G418/Puro-clones showed decreased expression of genes highly expressed in undifferentiated iPSCs after differentiation but increased expression of muscle-related genes. Interestingly, skeletal muscles derived from Puro-bulk iPSCs (YFE16, TIGE9, and EKN3) maintained in myotube differentiation medium for longer periods (3 days and 6 days) exhibited gene expression profiles more similar to those of Hu5/KD3-derived skeletal muscles than did those maintained in myotube differentiation medium for a shorter period (2 days), suggesting that longer culture in myotube differentiation medium promotes maturation of iPSC-derived skeletal muscles. Unfortunately, even hiPSC-derived skeletal muscles cultured in myotube differentiation medium for 1 week still exhibited expression profiles of skeletal muscle-related genes distinct from those of Hu5/KD3-derived skeletal muscles, suggesting that the cells were not sufficiently mature in this condition.

To determine the differences between skeletal muscles derived from Puro-bulk and G418-bulk or those derived from Puro-bulk and Puro-clones, we also performed gene set enrichment analysis (GSEA) based on the transcriptome data. Puro-bulk-derived skeletal muscles showed enrichment of skeletal muscle-related GO terms and pathways associated with myogenesis, muscle development, maturation, and contractile muscle fibers compared with those derived from G418-bulk or Puro-clones (Fig. 5b–e, Supplementary Fig. S8, Supplementary Tables S5–S12). These results suggest that Puro-bulk *MYOD1*-hiPSCs were capable of more efficiently differentiating into mature skeletal muscles than G418-bulk or Puro-clones.

Finally, principal component analysis (PCA) revealed that skeletal muscles derived from multiple Puro-bulk *MYOD1*-hiPSCs showed expression profiles that were closer to those of Hu5/KD3-derived skeletal muscles than undifferentiated hiPSCs and more closely resembled Hu5/KD3-derived skeletal muscles as the culture days progressed from day 2 to day 3 and day 6 after replacement with myotube differentiation medium (Fig. 5f). These processes were considered to indicate a transcriptional trajectory from undifferentiated iPSCs toward myogenic cells and mature myotubes (virtual myogenic timeline) and suggest that hiPSC-derived skeletal muscles are capable of differentiating into more mature skeletal muscles during long-term maturation culture. Furthermore, skeletal muscles derived from Puro-bulk 201B7-*MYOD1*-hiPSCs were located almost in the middle of skeletal muscles derived from 5 Puro-clones generated from the same hiPSC origin and showed an average expression profile. This, in combination with the results of qRT‒PCR analyses of muscle-related genes (Fig. 3b, Supplementary Fig. S5)^22^, suggests that Puro-bulk-derived skeletal muscles have the average properties of those derived from Puro-clones and that this approach may be beneficial for avoiding variation caused by clonal culture.

### SBMA disease-specific hiPSCs efficiently differentiated into mature skeletal muscles and showed reduced *ACTN3* expression, consistent with the decrease in fast-twitch muscle in SBMA patients

**Figure 6:**
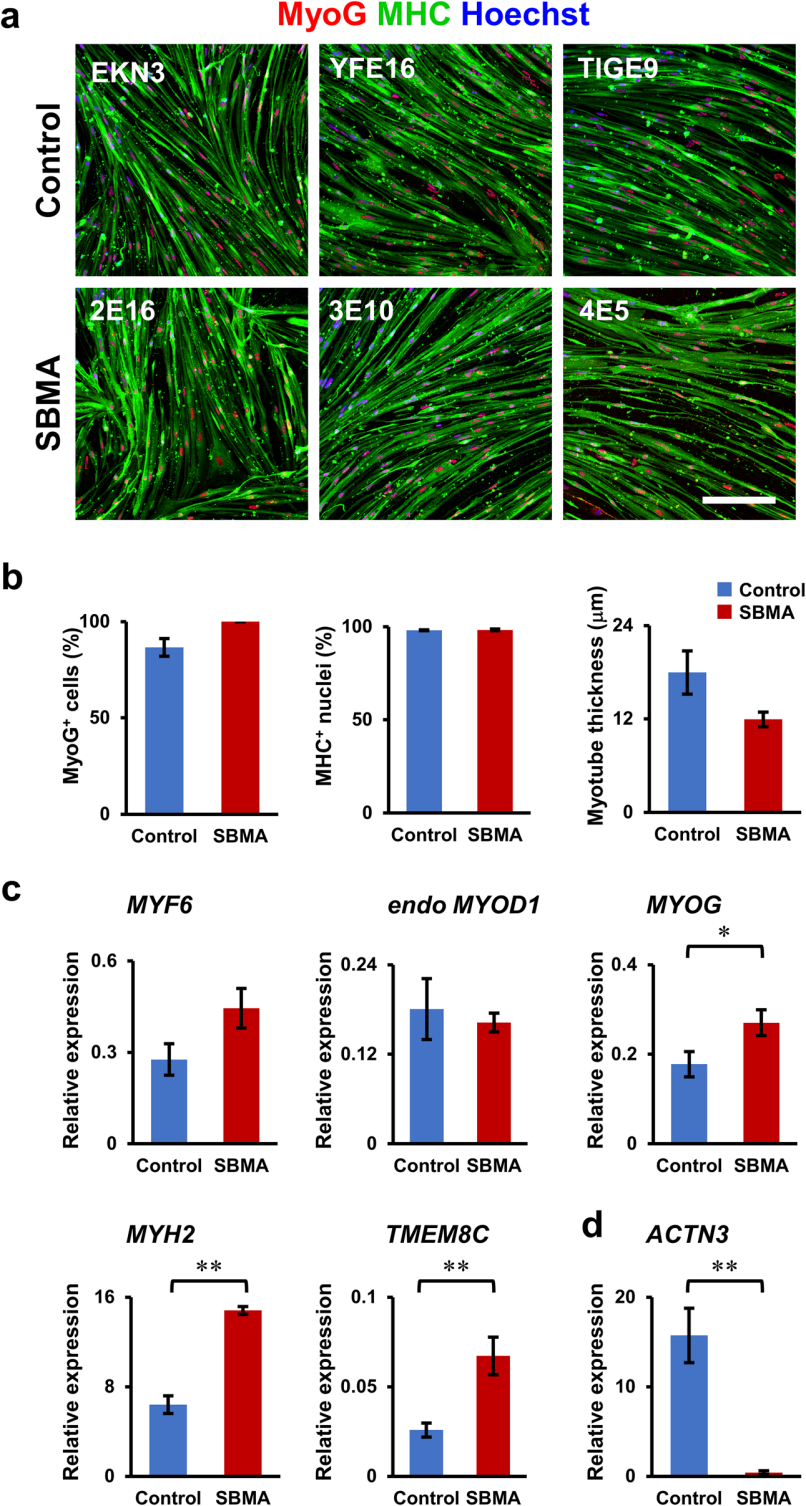
SBMA disease-specific hiPSCs efficiently differentiated into mature skeletal muscle and exhibited reduced *ACTN3* expression, consistent with the decrease in fast-twitch muscle in SBMA patients. (**a**) ICC analysis of MyoG (red) and MHC (green) in skeletal muscles derived from SBMA disease-specific hiPSCs and control hiPSCs. SBMA disease-specific hiPSCs could differentiate into mature skeletal muscles as efficiently as control hiPSCs. Scale bar, 200 μm. (**b**) Quantitative analysis of the parameters of skeletal muscle differentiation (MyoG^+^ cells and MHC^+^ nuclei) and maturation (thickness of myotubes). The data are presented as the mean ± SEM, n = 3. (**c**,**d**) Quantitative RT‒PCR analysis of SBMA and control hiPSC-derived skeletal muscles for *MYF6, endo-MYOD1, MYOG, MYH2,* and *TMEM8C*. (**c**) and *ACTN3* (**d**) expressed in fast-twitch muscles. The data are presented as the mean ± SEM, n = 9 (n = 3 each from 3 patients and 3 controls). *p < 0.05, **p < 0.01.

To investigate the applicability of our bulk differentiation system to disease analysis, we validated the differentiation of spinal bulbar muscular atrophy (SBMA) disease-specific hiPSCs into skeletal muscles. SBMA is an adult-onset, slowly progressive neuromuscular disease caused by abnormal expansion of the CAG repeat (polyglutamine tract) in the androgen receptor (AR) gene. Although SBMA has been considered a motor neuron disease, recent analyses have suggested that skeletal muscle is involved in its pathogenesis. SBMA disease-specific iPSCs (SBMA2E16, 3E10, and 4E5) and control iPSCs (TIGE9, YFE19, and EKN3)^28–30^ were transduced with the *Dox-3HA-hMYOD1 piggyBac* vector with a puromycin selection cassette and differentiated into skeletal muscles in bulk as described in Figs. 1b and 2a. ICC analysis at day 9 of differentiation revealed that both patient and control Puro-bulk *MYOD1*-hiPSCs efficiently and similarly differentiated into mature skeletal muscles, as confirmed by the proportions of MyoG^+^ cells, MHC^+^ nuclei, and myotube thickness of more than 85%, 95%, and 10 μm, respectively (Fig. 6a,b). Moreover, qRT‒PCR analysis of myogenic genes at day 9 of differentiation revealed that skeletal muscles derived from patient hiPSCs expressed sufficient levels of myogenic genes compared to those derived from control hiPSCs, and some of them, such as *MYOG*, *MYH2*, and *TMEM8C*, were more highly expressed in patient-derived muscles than in controls (Fig. 6c), consistent with a previous report showing an increase in myogenic gene expression in a mouse model of SBMA^32^. Interestingly, disease-specific hiPSC-derived skeletal muscles showed a significant decrease in the expression of *ACTN3*, a gene expressed in fast-twitch muscles, compared with that in controls (Fig. 6d), which supports previous reports showing a decrease in fast-twitch muscle in SBMA patients and mouse models^33^. Together, these results suggest that our highly efficient differentiation of Puro-bulk *MYOD1*-hiPSCs is applicable to the analysis of neuromuscular disorders.

### 3D muscle tissues were fabricated from Puro-bulk *MYOD1*-hiPSCs and exhibited contractility upon EPS

**Figure 7:**
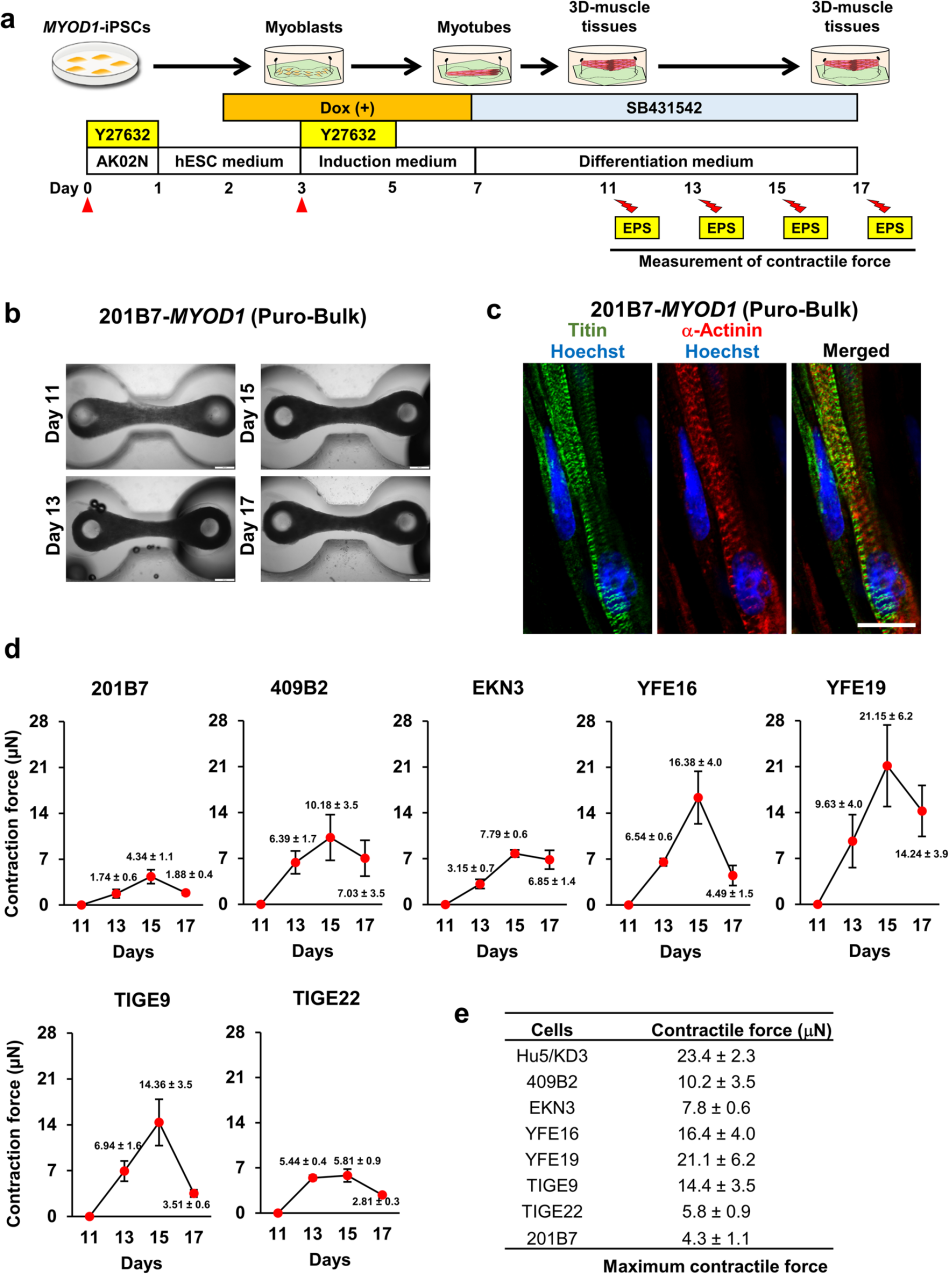
3D muscle tissues fabricated from Puro-bulk *MYOD1*-hiPSCs exhibited contractile force, indicating the functionality of the hiPSC-derived muscle tissues. (**a**) Schematic of the fabrication of 3D muscle tissues. On day 3 of differentiation, differentiating cells were dissociated and replated on microdevices. On day 11, muscle tissues were pulled up at the top of the pillar and processed for measurement of contractile force elicited by EPS at days 11, 13, 15 and 17. (**b**) Brightfield top-view images of muscle tissues derived from Puro-bulk 201B7-*MYOD1*-hiPSCs at days 11, 13, 15, and 17. Scale bar, 500 μm. (**c**) IHC analysis of fabricated muscle tissues from Puro-bulk 201B7-*MYOD1*-hiPSCs for Titin and α-Actinin at day 17 of differentiation. Sarcomere formation was clearly observed in Puro-bulk 201B7-*MYOD1*-hiPSC-derived muscle tissues. The nuclei were stained with Hoechst 33258. Scale bar, 20 µm. (**d**) Measurement of the contractile force of the muscle tissues derived from seven hiPSC clones (201B7, 409B2, EKN3, YFE16, YFE19, TIGE9, and TIGE22) at days 11, 13, 15, and 17 of differentiation. The maximum contractile force is recorded at day 15. The data are presented as the mean ± SEM, n = 4. (**e**) Comparison of the maximum contractile force of muscle tissues derived from seven hiPSC clones (201B7, 409B2, EKN3, YFE16, YFE19, TIGE9, and TIGE22) and Hu5/KD3 cells.

To investigate the functionality of skeletal muscles derived from bulk *MYOD1*-hiPSCs, 3D muscle tissues were fabricated from Puro-bulk *MYOD1*-hiPSCs established from seven hiPSC clones (201B7, 409B2, EKN3, YFE16, YFE19, TIGE9, and TIGE22) and examined for their contractility (Fig. 7a). According to the previously reported reseeding protocol^22^, all of these *MYOD1*-hiPSCs were differentiated into skeletal muscle cells by the same monolayer differentiation protocol until day 3 and were then redissociated and processed for the fabrication of 3D muscle tissues in the microdevices in the presence of 10 µM Y-27632. The cells were maintained in the presence of 1.5 µg/ml Dox from day 2 (before reseeding) up to day 7 (after reseeding) to induce skeletal muscle differentiation. All the *MYOD1*-hiPSCs gave rise to similar 3D muscle tissues that expressed α-Actinin and Titin at day 17 of differentiation according to immunohistochemical (IHC) analysis, which uncovered sarcomere structures and uniform alignment of striated muscle fibers (Fig. 7b,c, Supplementary Fig. S9). Time course gene expression analysis in 3D muscle tissue derived from Puro-bulk *MYOD1*-hiPSCs revealed that after the withdrawal of Dox at day 7, along with the decrease in *MYOD1* transgene expression, the expression of total *MYOD1* was decreased until day 15, and the expression of endogenous *MYOD1* remained low until day 13. Similarly, the expression of other myogenic genes was decreased or remained low, until day 13 for *MYF6*, *MYOG*, and *MYH*7 and until day 15 for *MYH2* and *TMEM8C*. Interestingly, all myogenic genes, including endogenous *MYOD1,* exhibited increased expression thereafter (Supplementary Fig. S10). On days 11, 13, 15, and 17, the contractile force of the fabricated muscle tissues was measured upon EPS (2 ms, 20–40 V, 30 Hz). On day 13 (day 10 of 3D culture), all the Puro-bulk *MYOD1*-hiPSC-derived muscle tissues exhibited contractile forces ranging from 1.7 ± 0.6 µN (201B7) to 9.6 ± 4.0 µN (YFE19) in response to EPS (Fig. 7d). On day 15 (day 12 of 3D culture), *MYOD1*-hiPSC-derived muscle tissues showed maximal contractile forces ranging from 4.3 ± 1.1 µN (201B7) to 21.1 ± 6.2 µN (YFE19), after which they decreased (Fig. 7d,e). The increase in contractile force after day 13 coincided with an increase in the expression of myogenic genes, suggesting a progression of maturation in this stage. Although all of the *MYOD*-hiPSC-derived 3D muscle tissues showed contractility, there was substantial variability among different hiPSC lines. Moreover, the maximum contractile force obtained in 3D muscle tissue generated from some *MYOD1*-hiPSCs, such as Puro-bulk YFE19-*MYOD1*-hiPSCs, was almost equivalent to the maximum contractile force shown by 3D skeletal muscle tissues fabricated from the human myoblast cell line Hu5/KD3, using the same devices and methods as reported previously^34^ (23.4 ± 2.3 μN at day 8 of 3D culture) (Fig. 7e). These results suggest that hiPSC-derived 3D muscle tissues generated by a simple method using Puro-bulk *MYOD1*-hiPSCs may have sufficient functionality to demonstrate contractility.

## Discussion

Our data suggest that for highly efficient skeletal muscle differentiation using bulk *MYOD1*-hiPSCs, the selection marker plays important roles in increasing transgene expression efficiency, which may ultimately result in increased differentiation efficiency. Although different selection markers are commonly speculated to affect transgene expression and the properties of transduced cells, there have been few reports describing the effects of selection markers, especially their effects on the differentiation properties of hPSCs. In our bulk culture differentiation, bulk *MYOD1*-hiPSCs established with puromycin selection showed similar or higher transgene expression and differentiation efficiency than clonally established *MYOD1*-hiPSCs. In contrast, G418-bulk *MYOD1*-hiPSCs, even those established with higher concentrations of G418, exhibited poor transgene expression and/or differentiation potential (Figs. 1f–i, 2, 3, 4, Supplementary Figs. S2c–f, S5–7). Notably, if we picked appropriate clones, clonally established *MYOD1*-hiPSCs selected with either G418 or puromycin achieved almost the same transgene expression and differentiation potential as control 409B2-*MYOD1*-hiPSCs. These data suggest that puromycin selection has the advantage of efficient transgene expression even for bulk *MYOD1*-hiPSCs compared with G418 selection and that sufficient transgene expression is key for high differentiation efficiency. Although the copy numbers of integrated transgenes may affect their expression levels, our results suggest that they do not necessarily correlate with the expression levels of transgenes or differentiation efficiency (Supplementary Figs. S2g and S5d). This could be due to a variety of factors, including silencing by epigenetic regulation.

Interestingly, bulk *MYOD1*-hiPSCs established with a higher concentration of puromycin (1.5 μg/ml), in which higher transgene expression could be achieved, did not show significantly greater differentiation efficiency than those established with a lower concentration of puromycin (0.5 μg/ml) (data not shown). Moreover, although bulk *MYOD1*-hiPSCs established from TIGE22 with a higher concentration of G418 expressed levels of transgenes as high as those of cells established from the same hiPSCs with puromycin, they did not differentiate into skeletal muscles as efficiently as the Puro-bulk line (Fig. 4, Supplementary Figs. S5–7). These findings suggest that not only the transgene expression level but also other factors, such as cell-to-cell variability in transgene expression, may affect the efficiency of myogenic differentiation from bulk *MYOD1*-hiPSCs. What, then, is responsible for the differences between G418 and puromycin? Both G418 and puromycin are aminoglycoside antibiotics that are commonly used for the selection of mammalian cells transduced with transgenes. Both G418 and puromycin inhibit protein synthesis by suppressing translation at ribosomes and inducing cell death. However, puromycin is distinct from G418 in that it induces cell death more quickly and powerfully^35,36^. In most cells, G418 exhibits relatively slow action, taking more than 1 week to gradually induce cell death, whereas puromycin may exhibit faster action to induce complete cell death within 2 to 4 days if the cells do not express resistance genes^35–37^.

Moreover, a previous analysis using HEK293 cells showed distinct effects on the levels of and cell-to-cell variability in transgene expression^38^. In that study, G418 was shown to induce lower expression of transgenes with higher cell-to-cell variability, whereas puromycin was shown to induce higher expression of transgenes (10 times more than G418) with lower cell-to-cell variability^38^. This difference might have been caused by the different stabilities and activities of the resistance genes. If the selection cassette for G418, *neo*^*r*^ from transposons (Tn601 or Tn5) that express aminoglycoside-3’-phosphotransferase (APH), is relatively stable and has relatively high antibiotic-inactivating activity, even the cells expressing relatively low levels of transgene and *neo*^*r*^ may survive after G418 selection, and cells expressing the transgenes at a variety of levels may remain. On the other hand, if the selection cassette for puromycin, *pac* (encoding puromycin N-acetyl-transferase), is relatively unstable and has relatively low antibiotic-inactivating activity, only cells expressing relatively high levels of the transgene and *pac* may survive after puromycin selection, and the transgene expression levels of the remaining cells may be relatively high with reduced cell-to-cell variability. In the abovementioned analysis using HEK293 cells, the level and uniformity of transgene expression were shown to be the highest with zeocin, relatively high with puromycin and hygromycin, and lowest with G418 and blasticidin S^38^. These results suggest that for efficient transduction of genes into hPSCs, zeocin, puromycin, or hygromycin may be the best choices to obtain bulk cells with increased transgene expression with reduced cell-to-cell variability or to increase the possibility of picking clones with higher transgene expression.

In addition, we achieved quick and efficient differentiation of bulk *MYOD1*-hiPSCs into skeletal muscles, which may have minimized the effects of clonal variations. To date, several methods have been reported that achieve bulk differentiation of hiPSCs into skeletal muscles using lentiviruses, adenoviruses, or *piggyBac* transposon vectors^4,14,15,18,20,25^. However, most of these differentiation protocols have never achieved high differentiation efficiency, short culture periods, and simple culture methods simultaneously. Flow cytometry and cell sorting may be useful for the purification of cells transduced with transgenes and have been used in several protocols^7,10,13^. However, the fluorescent reporter must be introduced into the cells in advance, and performing cell sorting each time is not appropriate as a simple skeletal muscle differentiation method applicable to pathological analysis using a large number of hiPSCs, which was the purpose of this study. Moreover, with these methods, researchers must begin with undifferentiated and unmodified hiPSCs for every differentiation, which causes considerable differentiation variability. In this study, bulk *MYOD1*-hiPSCs were established by a simple method: a single transfection of a *piggyBac* vector with transposase followed by selection with puromycin for 7 days (Fig. 1b, lower protocol). Since our method does not require additional procedures, such as FACS purification, it can be performed in settings where such instrumentation is not available. The reproducibility of the efficient differentiation from Puro-bulk *MYOD1*-hiPSCs was confirmed with at least seven clones of hiPSCs, and all the Puro-bulk *MYOD1*-hiPSCs achieved highly efficient skeletal muscle differentiation, resulting in more than 80% MyoG^+^ or MHC^+^ cells (Figs. 2b–d, 4b, Supplementary Fig. S6a,b). This percentage is significantly higher than those obtained with the previously reported bulk culture methods using *piggyBac* transposon vectors or adenoviruses, which have yielded approximately 40 to 50% MyoG^+^ cells and 50 to 70% MHC^+^ cells^14,18,25^. Moreover, in our method, once we generated *MYOD1*-hiPSCs, we did not have to return to using unmodified, undifferentiated hiPSCs, suggesting that we can easily reproduce differentiation at any time. More importantly, we confirmed that the Puro-bulk *MYOD1*-hiPSCs showed gene expression and differentiation efficiency similar to those of previously confirmed control 409B2-*MYOD1*-hiPSCs and conventional clonal *MYOD1*-hiPSCs (Fig. 2, Supplementary Fig. S3). Furthermore, transcriptome analysis revealed that Puro-bulk *MYOD1*-hiPSCs differentiated into mature skeletal muscles more efficiently than conventional clonal *MYOD1*-hiPSCs (Fig. 5 and Supplementary Fig. S8). Although the underlying mechanisms are not yet clear, the use of Puro-bulk *MYOD1*-hiPSCs may be beneficial not only because it is a simple method but also because it yields high-quality skeletal muscles. Mechanical stresses such as repeated passaging and cell stretching have been shown to reduce iPSC quality, which may have affected the differences between Puro-bulk and Puro-clones^39,40^.

In the time course gene expression analysis during skeletal muscle differentiation, the Dox-induced *MYOD1* transgene also induced endogenous *MYOD1* expression as described in the previous literature^16^, and the *MYOD1* transgene, endogenous *MYOD1*, and total *MYOD1* all reached their peak expression on day 5 and decreased thereafter (Fig. 3b, Supplementary Fig. S4). Most myogenic genes, including *MYH2* and *MYH7*, are regulated by *MYOD1*^41–44^, and when Dox induced expression of *MYOD1* above physiological levels, significant myogenic gene expression was induced in response. Subsequent removal of Dox on day 7 resulted in loss of the *MYOD1* transgene and maintenance of only endogenous *MYOD1*. As a result, the expression of myogenic genes was decreased to and maintained at physiological levels, which may be involved in the maturation and functionality of the induced skeletal muscle.

More interestingly, we found that Puro-bulk *MYOD1*-hiPSCs exhibited properties comparable to the average properties of clonally established *MYOD1*-hiPSCs of the same origin, which showed considerable variability among clones, not only by gene expression analysis of skeletal muscle-related genes (Fig. 3b and Supplementary Fig. S4) but also by global transcriptome analyses, such as PCA (Fig. 5f). *MYOD1*-hiPSCs established in bulk culture are considered to have intercellular heterogeneity, resulting in a mixture of cells with varying levels of transgene expression. Clonally established *MYOD1*-hiPSCs are cloned from a heterogeneous cell population in bulk culture, and the cell-to-cell variability of the original bulk-*MYOD1* hiPSCs should be more clearly expressed as clone-to-clone variability, known as clonal variation^45,46^. In contrast, bulk *MYOD1*-hiPSCs, when established by puromycin selection, are established as a relatively homogenous population expressing high levels of transgene (Figs. 1f–i, 4a, Supplementary Figs. S2b–f, S5a–c), thus achieving highly efficient and homogenous skeletal muscle differentiation, which is expected to result in an average phenotype of the included cells. In the past, the variability of hiPSC clones has been known to mask phenotypes and drug efficacy in disease analyses. In many cases, multiple hiPSC clones had to be established and analyzed, which was a very laborious process, and the use of bulk hiPSCs instead of clonally established hiPSCs has been discussed^45–47^. However, as our bulk *MYOD1*-hiPSCs showed averaged phenotypes of multiple clones, therefore requiring only one bulk *MYOD1*-hiPSC line for one hiPSC clone, they may save valuable time and labor, enable the analysis of large numbers of hiPSC clones within short periods, and provide valuable in vitro skeletal muscle models, especially for analyses using disease-specific hiPSCs. The use of SBMA disease-specific hiPSCs in the current analysis supports the applicability of this method to disease analysis. In addition, the advantage of bulk culture has also been shown in the establishment of disease-specific hiPSCs of sporadic amyotrophic lateral sclerosis (ALS) and in an analysis of motor neurons derived from them. A collection of a large number of patient-derived lymphoblastoid cells from a multicenter ALS cohort in Japan, named the Japanese Consortium for Amyotrophic Lateral Sclerosis Research (JaCALS), was established for hiPSCs in a bulk manner. The cells were subsequently differentiated into motor neurons and exhibited differentiation efficiencies and phenotypes similar to those of corresponding clonally established hiPSCs. The phenotypes also reflected those observed in the corresponding ALS patients^48^. Therefore, myogenic differentiation of bulk *MYOD1*-hiPSCs with our system may be particularly beneficial for analyses of sporadic diseases, which require analyses of large numbers of patient-derived hiPSCs.

Notably, we could have derived mature and functional skeletal muscles from bulk *MYOD1*-hiPSCs. Sarcomere formation was clearly shown by the expression of α-Actinin and Titin in monolayer myotubes, and 3D muscle tissues were fabricated from Puro-bulk *MYOD1*-hiPSCs^8,31,49^ (Fig. 7b,c, Supplementary Figs. S7d, S9). Moreover, all the fabricated 3D muscle tissues exhibited contractile force upon EPS (maximum contractile force ranging from 4.3 to 21.1 μN; Fig. 7d,e). This force was comparable to or higher than the contractile force observed in previously reported hiPSC- or human myoblast cell line (Hu5/KD3)-derived 3D muscle tissues fabricated using similar microdevices^34,50^ or equivalent to the contractile force observed in 3D muscle tissues fabricated by different types of microdevices, considering the differences in the cell number and microdevices used for the analysis^51^, suggesting the functionality of the muscle tissues derived from bulk *MYOD1*-hiPSCs. Since the skeletal muscle differentiation shown in this study was achieved by forced expression of *MYOD1*, which skips several important developmental processes, it is extremely important to demonstrate the functionality of the induced skeletal muscle by the contractility of 3D muscle tissues. In some of the hiPSCs, the observed contractile force was still lower than that observed in other hiPSCs or Hu5/KD3 cells (Fig. 7e)^34^. In addition, all the hiPSC-derived 3D muscle tissues showed a decrease in contractile force after day 15 for unknown reasons. Thus, the differentiation system still needs to be optimized to produce more mature and contractible muscle tissues for further analysis.

By minimizing the effects of clonal variations and generating contractible muscle tissues, our skeletal muscle differentiation system using Puro-bulk *MYOD1*-hiPSCs will provide a new approach for disease-specific hiPSC studies. This approach may facilitate the generation of better disease models for pathophysiological analysis of muscular disorders, especially those in which large numbers of hiPSCs derived from a large number of patients and controls are considered necessary.

## Materials and methods

### Construction of a Dox-inducible *3HA-hMYOD1*-expressing *piggyBac* vector

Human *MYOD1* (*hMYOD1*) cDNA was cloned and inserted into the pENTR vector and tagged with 3× HA at the N-terminus. Then, *3HA-hMYOD1* cDNA was transferred to PB-TA-ERN (Addgene #80474) or PB-TA-ERP2 (Addgene #80477)^52^ (kindly provided by Dr. Knut Woltjen, Kyoto University) with Gateway cloning technology to generate *piggyBac* vectors for Dox-inducible expression of *3HA-hMYOD1 (Dox-3HA-hMYOD1* vector), PB-TA-*3HA-hMYOD1*-ERN (for selection with G418) and PB-TA-*3HA-hMYOD1*-ERP2 (for selection with puromycin).

### Human iPSC culture and *MYOD1*-iPSC establishment

The hiPSCs used in this study were established previously (EKN3, YFE16, YFE19, TIGE9, TIGE22)^28–30^ or kindly provided by Dr. Shinya Yamanaka, Kyoto University, Japan (201B7 and 409B2)^2,53^. SBMA disease-specific hiPSCs were established previously (SBMA2E16, 3E10, and 4E5)^30^. The hiPSCs were grown on mitomycin C-treated SNL murine fibroblast feeder cells on gelatin-coated (0.1%) tissue culture dishes and were maintained in standard hESC medium consisting of Dulbecco’s modified Eagle’s medium (DMEM)/F12 supplemented with 20% KnockOut™ serum replacement (KSR; Thermo Scientific, USA), nonessential amino acids, 0.1 mM 2-mercaptoethanol (2-ME; Sigma‒Aldrich, USA), and 4 ng/ml FGF-2 (PeproTech, USA) at 37 °C in a humidified atmosphere containing 3% CO_2_. For feeder-free culture of hiPSCs, hiPSCs maintained on SNL feeder cells were transferred to plates coated with iMatrix-511 silk (Nippi, Japan) and maintained in StemFit medium (AK02N, Ajinomoto, Japan) at 37 °C in a humidified atmosphere containing 5% CO_2_.

To generate *MYOD1*-hiPSCs, semiconfluent hiPSCs maintained under feeder-free conditions were dissociated into single cells by Accutase (Nacalai Tesque, Japan), and 1.25 × 10^5^ cells were electroporated with 0.5 μg of PB-TA-*3HA-hMYOD1*-ERN (with G418 selection cassette) or PB-TA-*3HA-hMYOD1*-ERP2 (with puromycin selection cassette) in combination with 0.5 μg of transposase expression vector (CAG-hyPBase) via a Neon transfection system (Thermo Fisher Scientific, USA) with a 10 μl Neon tip using condition #6 (pulse voltage 1100 V, pulse width 30 ms, pulse no. 1). Then, the cells were plated onto 6-well plates in StemFit medium (AK02N) containing 10 μM Y27632 (Wako, Japan). Two days after transfection, the medium was replaced with fresh medium containing 100 µg/mL G418 (Wako, Japan) or 0.5 µg/mL puromycin (Sigma‒Aldrich, USA) and cultured for up to 7 days until hiPSC colonies appeared. The medium was changed every other day or every 2 days. To establish clonal *MYOD1*-hiPSCs (G418-clones, Puro-clones), 12 *MYOD1*-hiPSC colonies were picked from each of the G418- or puromycin-selected plates and expanded in the presence of G418 or puromycin. Then, 6 *MYOD1*-hiPSC clones that were morphologically better maintained in an undifferentiated state and proliferated better than other clones were selected from each Puro-clone and G418-clone and further expanded for up to 2 weeks to prepare frozen stocks (Fig. 1b upper panel). To generate bulk *MYOD1*-hiPSCs (G418-bulk line, Puro-bulk line), G418- or puromycin-treated *MYOD1*-hiPSC colonies were bulk-passaged and expanded in the presence of 100 µg/mL G418 or 0.5 µg/mL puromycin, respectively, to prepare frozen stocks (Fig. 1b lower panel). 409B2-*MYOD1*-hiPSCs were established previously via transduction of PB-TA-*hMYOD1*-ERP^22^ and were used as control *MYOD1*-hiPSCs. These *MYOD1*-hiPSCs were confirmed to efficiently differentiate into skeletal muscles.

### Differentiation of hiPSCs into skeletal muscles

For differentiation into skeletal muscles, semiconfluent *MYOD1*-hiPSCs maintained under feeder-free conditions were dissociated into single cells by Accutase and plated onto 24-well plates coated with growth factor-reduced Matrigel for 2 h at a dilution of 1:50 (Corning, USA) at a density of 7 × 10^4^ cells/well in StemFit medium (AK02N) with 10 μM Y27632. On day 1, the medium was changed to hESC medium without FGF-2. On day 3, the medium was replaced with skeletal muscle induction medium consisting of αMEM (Nacalai Tesque, Japan) supplemented with 10% KSR, 2% Ultroser G (BioSepra, Pall, USA), and 0.1 mM 2-ME. To induce the expression of *3HA-hMYOD1*, 1.5 μg/ml Dox (Wako, Japan) was added to the medium from day 2 to day 6 or 7. The medium was changed every other day. On day 6 or 7, for the formation of mature myotubes, the medium was substituted with myotube differentiation medium consisting of DMEM (high glucose, 1500 mg/L NaHCO_3_, Kohjin Bio, Japan) supplemented with 5% horse serum (HS; Sigma‒Aldrich, USA), 10 ng/mL recombinant human insulin-like growth factor 1 (IGF-1) (R&D Systems, USA), and 10 μM SB431542 (Tocris, UK). The medium was changed every 2 days, and the cells were maintained for an additional 2, 3, or 6 days for the maturation of myotubes.

### Culture of Hu5/KD3 cells

The Hu5/KD3 cells were maintained and differentiated as previously described with modifications^54–56^. Briefly, the cells were cultured in DMEM (Nacalai Tesque, Japan) supplemented with 20% FBS (Nichirei, Japan) and 2% Ultroser G (BioSepra, Pall, USA) at 37 °C in a humidified atmosphere containing 5% CO_2_. Passaging was conducted when the cells reached 80–90% confluency.

### Fabrication of 3D-muscle tissues from *MYOD1*-iPSCs

Fabrication of 3D muscle tissues on microdevices and measurement of contractile force were performed as described previously with modifications^34,57^. Briefly, microdevices made of polydimethylsiloxane (PDMS; Silpot 184, Dow Corning, Toray, Japan) were installed at the center of each well of a 48-well plate, sterilized under UV light for more than 1 h, and coated with 2% Pluronic F-127 solution (Thermo Fisher Scientific, USA) for 1 h at room temperature to prevent hydrogel adhesion.

Semiconfluent human *MYOD1*-hiPSCs maintained under feeder-free conditions were dissociated into single cells and differentiated into muscle cells on 6-well plates at a density of 3.5 × 10^5^ cells/well following the same protocol used for monolayer differentiation. From day 2, the expression of the *3HA-hMYOD1 or MYOD1* transgene was induced with 1.5 μg/ml Dox. On day 3, the cells were redissociated by Accutase^22^, resuspended in skeletal muscle induction medium, and processed for the fabrication of 3D muscle tissues. A hydrogel mixture was prepared by mixing ice-cold fibrinogen from bovine plasma (10 mg/mL, F8630, Sigma‒Aldrich, USA), Matrigel (Corning, USA), and 2 × DMEM with a volume ratio of 0.2:0.1:0.2. Then, the cell suspension (6 × 10^6^ cells/ml) and the hydrogel mixture were mixed at a volume ratio of 0.484:0.5. Finally, thrombin from bovine plasma (50 U/ml, Sigma‒Aldrich, USA) was added to the mixture at a thrombin:total mixture volume ratio of 0.016:1. Forty-five microliters of the resulting cell and hydrogel solution mixture (1.3 × 10^5^ cells) was poured into the dumbbell-shaped pocket on each microdevice, and the plates were incubated at 37 °C to solidify the hydrogel. The cells applied to the devices were cultured for 4 days in skeletal muscle induction medium with 0.5% penicillin/streptomycin (P/S) in the presence of 1.5 μg/ml Dox^27^. Y27632 (10 μM) was added to the medium for the first 2 days (from day 3 to day 5) of the 3D-muscle culture. The medium was changed every 2 days. On day 7, the medium was switched to differentiation medium consisting of DMEM, 5% HS, 10 μM SB431542, and 0.5% P/S without Dox, which was changed every other day.

For the fabrication of 3D muscle tissues from Hu5/KD3 cells, semiconfluent myoblast cells were dissociated by using 0.05% trypsin–EDTA, resuspended in growth medium (GM) consisting of DMEM supplemented with 10% FBS, and processed as reported previously^57^. The cell suspension (2 × 10^6^ cells/ml) and the hydrogel mixture were mixed following the same protocol used for the hiPSCs. The tissues were cultured in GM for 2 days, and the medium was then switched to differentiation medium consisting of DMEM supplemented with 2% HS, 1% P/S, and 1% insulin-transferrin-selenium (ITS) (Sigma‒Aldrich, USA). On day 6, muscle tissues were pulled up at the top of the pillar and processed for the measurement of contractile force after EPS.

All the culture media contained 2.0 mg/mL 6-aminocaproic acid (6AA, Sigma‒Aldrich, USA) and 1 mg/ml trans-4-aminomethyl cyclohexane carboxylic acid (TA, Tokyo Chemical Industries, Japan) to prevent disassembly of the tissues.

### Contractile force measurement of 3D muscle tissues

The contractile force of the 3D muscle tissues was measured as reported previously^34^. Muscle tissues were electrically stimulated to achieve maximum tetanic force with an electrical stimulus of 4.0 V/mm at 30 Hz with 2 ms wide pulses (C-Pace EP, IonOptix, USA) using customized electrodes. Electrical stimulation was applied twice to each tissue sample for 5 s on days 11, 13, 15, and 17, and the displacement of the tips of the microposts was observed with an upright microscope (BX53F, Olympus, Japan or AxioImager M2, Carl Zeiss, Germany) under a 4× objective (UPLFN4X) or a 20× objective (SLMPLN20X). The displacement of the tip of each micropost was measured with ImageJ software using photographs taken with and without stimulation, and the average value from both microposts was obtained. The contractile force (F) was determined by the equation F = 3πER^4^δ/(4L^3^), where E is the elastic modulus of PDMS (1.7 MN/m^2^), R is the radius of a micropost (0.25 mm), δ is the displacement of the tip of the micropost, and L is the length of the micropost (4 mm)^58^.

### Histological analysis

ICC and IHC analyses of cultured cells and 3D muscle tissues, respectively, were performed as described previously with modifications^34,59^. Cultured cells were fixed with 4% paraformaldehyde (PFA) for 15 min at room temperature. After blocking in blocking buffer consisting of phosphate-buffered saline (PBS) containing 10% FBS and 0.3% Triton X-100 for 1 h at room temperature, the cells were incubated with primary antibodies at 4 °C overnight (see Supplementary Table S2 for the primary antibodies). After three washes with PBS, the cells were incubated with secondary antibodies conjugated with Alexa Fluor 488, Alexa Fluor 555, or Alexa Fluor 647 for 1 h at room temperature. For the quantitative analysis, photographs of five randomly selected visual fields were taken and used for quantification of the immunopositive cells and nuclei and measurement of the immunopositive area and myotube thickness manually or with ImageJ software^60^.

Muscle tissues were fixed with 4% PFA for 30 min at room temperature. After permeabilization with PBS containing 0.3% Triton X-100 for 10 min, the tissues were incubated in blocking buffer consisting of PBS containing 10% goat serum and 0.01% Triton X-100 for 30 min at room temperature and then incubated with primary antibodies (see Supplementary Table S2 for the primary antibodies) for 3 h at room temperature. After three washes with PBS, the cells were incubated with secondary antibodies conjugated with Alexa Fluor 488 or Alexa Fluor 555 for 2 h at room temperature.

Both the cells and the muscle tissues were stained for nuclei with 10 μg/ml Hoechst 33258 (Sigma‒Aldrich, USA) and were observed with a confocal laser scanning microscope (LSM700 or LSM900, Carl Zeiss, Germany).

### Genomic DNA isolation and qPCR analysis

Genomic DNA was isolated using GenePrepStar (PI-498α, Kurabo) according to the manufacturer’s instructions and then processed for qPCR analysis using the primers for *HA-hMYOD1* transgenes and *β-ACTIN*. The primer sequences and cycling conditions are indicated in Supplementary Table S3.

### RNA isolation and qRT‒PCR analysis

RNA was isolated using an RNeasy Mini Kit (Qiagen, Germany) and then converted into cDNA using PrimeScript RT Master Mix (Takara Bio Inc., Japan). Real-time qRT‒PCR was performed as previously described using TB Green Premix Ex Taq II (Takara Bio Inc, Japan) and a QuantStudio 7 Real-Time PCR system (Thermo Fisher Scientific, USA)^61^. The amount of cDNA was normalized to that of human-specific *β-ACTIN* mRNA. The primer sequences and PCR cycling conditions are listed in Supplementary Table S1.

### RNA sequencing and data analysis

For all samples, RNA-seq libraries were constructed by using total RNA and prepared according to the Strand-Specific Transcriptome Library Construction Protocol or BGISEQ-500 RNA-Seq Library Preparation Protocol (DNBSEQ). The libraries were subsequently sequenced on a DNBSEQ-G400 (MGI) according to the manufacturer’s instructions. The reads were aligned to the reference human genome (hg38) with STAR (ver.2.7.5a) software (https://github.com/alexdobin/STAR)^62^^.^ The reads aligned to rRNA and transfer RNA (tRNA) regions were removed. The mapped reads were assigned to genes (Ensembl database annotation version GRCh38.100) using FeatureCounts from the Bioconductor package Rsubread (ver.2.0.1)^63^. Normalization and differential expression analysis were performed using DESeq2 (ver.1.26.0)^64^.

The expression data were grouped using a hierarchical clustering algorithm (ward. D2) by average linkage with the Pearson distance and visualized by ComplexHeatmap (ver. 2.13.1) (https://github.com/jokergoo/ComplexHeatmap)^65^ and dendsort (ver. 0.3.4) (https://github.com/evanbiederstedt/dendsort)^66^. GSEA^67^ was performed by using GSEA 4.2.3 to identify Gene Ontology (GO) terms and pathways associated with altered gene expression for each of the comparisons. The gene sets were downloaded from the Molecular Signatures Database (MSigDB), and C2 (curated gene sets: chemical and genetic perturbation (CGP) and canonical pathway (CP)) and C5 (GO gene sets) were used for GSEA. PCA was performed using TCC-GUI (https://github.com/swsoyee/TCC-GUI)^68^.

Genes with significantly altered expression between undifferentiated *MYOD1*-hiPSCs (day 0) and control iPSC (409B2-*MYOD1*)-derived skeletal muscles (day 9) or human myoblast cell line (Hu5/KD3)-derived skeletal muscles, which were used for hierarchical clustering, were identified. Among a total of 60,624 transcripts detected by RNA sequencing, 22,092 transcripts were identified with a feature count of 10 or more in all 3 samples of undifferentiated iPSCs (day 0) or in all 3 samples of Hu5/KD3-derived skeletal muscles and with a feature count of 10 or more either in all 3 samples in undifferentiated iPSCs (day 0) or in a sample of skeletal muscles derived from 409B2-*MYOD1* hiPSCs on day 9. Then, 16,583 transcripts with a statistically significant difference between undifferentiated iPSCs (day 0) and Hu5/KD3-derived skeletal muscles (*Padj* < 0.05) were selected. Finally, 2795 transcripts (2794 genes) with feature counts of more than twice (1960 transcripts (1960 genes)) or less than half (835 transcripts (834 genes)) in both Hu5/KD3-derived skeletal muscles and 409B2-*MYOD1* hiPSC-derived skeletal muscles compared to undifferentiated hiPSCs were identified. The identified genes are listed in Supplementary Table S4.

### Statistical analysis

All the data were collected from three independent cultures. Statcel4 software (OMS Publishing Inc., Saitama, Japan) was used for the statistical analyses. For all experiments, the data are presented as the mean ± standard error of the mean (SEM). For comparisons among the groups, statistical analyses were performed by one-way analysis of variance (ANOVA) followed by a post hoc Bonferroni test. A *p* value less than 0.05 was considered to indicate statistical significance. For the differential analysis of mRNAs between groups using DESeq2, an adjusted *p* value for false discovery rate (FDR) correction was obtained with the Benjamini–Hochberg (B-H) method.

### Study approval

All experimental procedures involving the use of hiPSCs were approved by the ethics committee of the Aichi Medical University School of Medicine (approval numbers 14-004, 2020-213, and 2022-431).

## Supplementary Information

Supplementary Legends.

Supplementary Figure S1.

Supplementary Figure S2.

Supplementary Figure S3.

Supplementary Figure S4.

Supplementary Figure S5.

Supplementary Figure S6.

Supplementary Figure S7.

Supplementary Figure S8.

Supplementary Figure S9.

Supplementary Figure S10.

Supplementary Tables.

## Data Availability

The datasets supporting the conclusions of this article are available in the following repositories: GEO (RNA sequence expression data, http://www.ncbi.nlm.nih.gov/geo/, Accession number: GSE212366).
