# Supplementary material for: Simple and efficient differentiation of human iPSCs into contractible skeletal muscles for muscular disease modeling
Source: Sci Rep. 2023 May 25;13:8146. doi: 10.1038/s41598-023-34445-9 (PMC10213064; doi:10.1038/s41598-023-34445-9)
Supplement: Supplementary file 1 — Supplementary Legends. [file 41598_2023_34445_MOESM1_ESM.docx]

**Supplementary Information**

**Simple and efficient differentiation of human iPSCs into contractible skeletal muscles for muscular disease modeling**

Muhammad Irfanur Rashid^1,2^, Takuji Ito^1,2,3^, Fuyuki Miya^4^, Daisuke Shimojo^2,5^, Kanae Arimoto^6^, Kazunari Onodera^1,2,7^, Rina Okada^1,2,3^, Takunori Nagashima^6^, Kazuki Yamamoto^6^, Zohora Khatun^1,2^, Rayhanul Islam Shimul^1^, Jun-ichi Niwa^2^, Masahisa Katsuno^7,8^, Gen Sobue^9^, Hideyuki Okano^5^, Hidetoshi Sakurai^10^, Kazunori Shimizu^6^, Manabu Doyu^2^, Yohei Okada^1,2,^*

^1.^ Department of Neural iPSC Research, Institute for Medical Science of Aging, Aichi Medical University, 1-1 Yazakokarimata, Nagakute, Aichi, 480-1195, Japan

^2.^ Department of Neurology, Aichi Medical University School of Medicine, 1-1 Yazakokarimata, Nagakute, Aichi, 480-1195, Japan

^3.^ Japan Society for the Promotion of Science, 5-3-1 Kojimachi, Chiyoda-ku, Tokyo, 102-0083, Japan

^4.^ Center for Medical Genetics, Keio University School of Medicine, 35 Shinanomachi, Shinjuku-ku, Tokyo, 160-8582, Japan

^5.^ Department of Physiology, Keio University School of Medicine, 35 Shinanomachi, Shinjuku-ku, Tokyo, 160-8582, Japan

^6.^ Department of Biomolecular Engineering, Graduate School of Engineering, Nagoya University, Furo-cho, Chikusa-ku, Nagoya, Aichi, 464-8603, Japan

^7.^ Department of Neurology, Nagoya University Graduate School of Medicine, Showa-ku, Nagoya, Aichi, 466-8650, Japan

^8.^ Department of Clinical Research Education, Nagoya University Graduate School of Medicine, Showa-ku, Nagoya, Aichi, 466-8650, Japan

^9.^ Aichi Medical University, 1-1 Yazakokarimata, Nagakute, Aichi, 480-1195, Japan

^10.^ Department of Clinical Application, Center for iPS Cell Research and Application (CiRA), Kyoto University, 53 Kawahara-cho, Shogoin, Sakyo-ku, Kyoto, 606-8507, Japan

***Correspondence:**

Yohei Okada

Department of Neural iPSC Research, Institute for Medical Science of Aging, Aichi Medical University

Address: 1-1 Yazakokarimata, Nagakute, Aichi, 480-1195, Japan

E-mail: [yohei@aichi-med-u.ac.jp](mailto:yohei@aichi-med-u.ac.jp); Phone: +81-561-76-3353; Fax: +81-561-76-0868

**Supplementary Figure Legends**

**Supplementary Fig. S1 Expression of pluripotent stem cell markers in *MYOD1*-hiPSCs.**

a, b. ICC analysis of the clonal *MYOD1*-hiPSCs established with G418 (a) or puromycin selection (b) for pluripotent stem cell markers (Oct3/4 and Nanog). The nuclei were stained with Hoechst 33258. All *MYOD1*-hiPSCs retained the expression of pluripotent stem cell markers. Scale bar, 50 μm.

**Supplementary Fig. S2 Dox-inducible expression of *3HA-hMYOD1* under undifferentiated conditions.**

a. Schematic of the analysis of transgene expression under undifferentiated conditions with or without Dox. *MYOD1*-hiPSCs were cultured in the presence of Dox from day 2 to day 5 or in the absence of Dox under undifferentiated conditions. Samples were collected at the times indicated by the closed triangles.

b-e. ICC analysis of the expression of transgenes (HA) and MyoD1 in cells cultured without Dox (b) or with Dox (c). Scale bar, 200 μm. Quantitative analyses of HA^+^ and MyoD1^+^ cells are shown in d and e, respectively. The G418-bulk line exhibited lower expression of transgenes (HA) and MyoD1 than the G418-clones, whereas the Puro-bulk line showed expression of transgenes (HA) and MyoD1 similar to that of the Puro-clones.

f. Transgene expression in bulk and clonal *MYOD1*-hiPSCs established with G418 or puromycin selection in the presence of Dox as examined by qRT‒PCR and compared with that in control 409B2-*MYOD1*-hiPSCs. The amount of cDNA was normalized to that of human-specific *β-ACTIN*.

The data are presented as the mean ± SEM, n = 3. *, *p < 0.05*, **, *p < 0.01*. ANOVA followed by *post hoc* Bonferroni test.

g. Relative copy numbers of integrated transgenes in the genomic DNA of bulk and clonal 201B7-*MYOD1*-hiPSCs established with G418 or puromycin selection as examined by qPCR. The amount of genomic DNA was normalized to that of *β-ACTIN*. The data are presented as the mean ± SEM.

**Supplementary Fig. S3 Time course of myogenic differentiation of clonal *MYOD1*-hiPSCs established with G418 or puromycin selection.**

a, b. ICC analysis of myotubes derived from clonal *MYOD1*-hiPSCs established by G418 or puromycin selection for the expression of MyoG and MHC at day 9 of differentiation. The nuclei were stained with Hoechst 33258. Scale bar, 200 μm.

c, d. Brightfield images showing the time course of myogenic differentiation of the established clonal *MYOD1*-hiPSCs with G418 selection. All clonal *MYOD1*-hiPSCs morphologically differentiated into myoblasts, subsequently formed myotubes, and achieved a myotube-like aligned structure by day 7. Scale bar, 100 μm.

**Supplementary Fig. S4 Time course gene expression analysis in bulk and clonal *MYOD1*-hiPSCs with G418 or puromycin selection.**

Time course gene expression analysis of bulk and clonal *MYOD1*-hiPSCs with G418 or puromycin selection as well as control 409B2-*MYOD1*-hiPSCs along with myogenic differentiation. Compared with the Puro-clones and 409B2-*MYOD1*-hiPSCs, the Puro-bulk line showed similar or average expression, whereas the G418-bulk line showed lower expression levels than other *MYOD1*-hiPSCs throughout the differentiation process. Moreover, in contrast to the large clonal variations observed in differentiating Puro clones, the Puro-bulk line showed average expression of the six Puro clones or expression similar to that in the G418 clones for all the genes associated with skeletal muscle differentiation during the differentiation processes. The amount of cDNA was normalized to that of human-specific *β-ACTIN* and is presented as the expression relative to that in undifferentiated hiPSCs (*NANOG* and *OCT3/4*), the human myoblast cell line Hu5/KD3 differentiated for 3 days (*CD56*, total and endogenous *MYOD1*, *MYOG*, *MYF6*, *MEF2C*, *MYH2*, *MYH7*, and *TMEM8C*), and EKN3-*MYOD1* iPSCs differentiated for 5 days (*Tg MYOD1*). The data are presented as the mean ± SEM, n = 3. *, *p < 0.05*, **, *p < 0.01*.

**Supplementary Fig. S5 Bulk *MYOD1*-iPSCs established by puromycin selection showed higher transgene expression than those established by G418 selection.**

a. ICC analysis of the expression of transgenes (HA) and MyoD1 in cells cultured under differentiating conditions in the presence of Dox. Scale bar, 200 μm.

b, c. Quantitative analyses of HA^+^ and MyoD1^+^ cells are shown in b and c, respectively. The expression levels of transgenes (HA) and MyoD1 were lower in the G418-bulk line at any concentration than in the Puro-bulk line.

d. Relative copy numbers of integrated transgenes in the genomic DNA of bulk *MYOD1*-hiPSCs (201B7, 409B2, EKN3, YFE16, YFE19, TIGE9, TIGE22) established by selection with puromycin or various concentrations of G418 as examined by qPCR. The amount of genomic DNA was normalized to that of *β-ACTIN*. The data are presented as the mean ± SEM, n = 3. *, *p < 0.05*, **, *p < 0.01*. ANOVA followed by *post hoc* Bonferroni test.

**Supplementary Fig. S6 Bulk *MYOD1*-hiPSCs established by puromycin selection showed more efficient differentiation potential than those obtained by G418 selection.**

a, b. Quantitative analysis of the parameters for skeletal muscle differentiation, including the proportions of MyoG^+^ cells among total cells (a) and the proportions of MHC^+^ nuclei among total nuclei (b). All the Puro-bulk *MYOD1*-hiPSCs exhibited higher differentiation potential than the *MYOD1*-hiPSC lines generated by selection with different concentrations of G418.

c-e. The expression of *MYH7, MYF6,* and *MYOG* in bulk *MYOD1*-hiPSC lines established from seven hiPSC clones by selection with puromycin (0.5 μg/ml) or various concentrations of G418 (100 μg/ml, 300 μg/ml, 500 μg/ml) at day 9 of differentiation. The amount of cDNA was normalized to that of human-specific *β-ACTIN* and is presented as the relative expression in the human myoblast cell line Hu5/KD3 differentiated for 3 days. The data are presented as the mean ± SEM, n = 3. *, *p < 0.05*, **, *p < 0.01*. ANOVA followed by *post hoc* Bonferroni test.

**Supplementary Fig. S7 Bulk *MYOD1*-hiPSCs established by puromycin exhibited differentiation into more mature myotubes than those established by G418 selection.**

a-c. Quantitative analysis of the parameters for myotube maturation, including the myotube thickness (a), number of nuclei per myotube (b), and MHC^+^ area per myotube (c). All the Puro-bulk *MYOD1*-hiPSCs exhibited myotube maturation potential higher than that of G418-bulk *MYOD1*-hiPSCs. The data are presented as the mean ± SEM, n = 3. *, *p < 0.05*, **, *p < 0.01*. ANOVA followed by *post hoc* Bonferroni test.

d. ICC analysis indicating the maturation of all the Puro-bulk *MYOD1*-hiPSC-derived myotubes according to the sarcomere structure as shown by α-actinin staining. The arrowheads indicate sarcomere formation. Scale bar, 50 μm.

**Supplementary Fig. S8 GO and pathway GSEA between Puro-bulk and Puro-clones**

1. GO enrichment analysis. The top 10 upregulated gene sets in Puro-bulk and Puro-clones with *FDR q*-values < 0.01 are shown. The red bar indicates muscle-related gene sets, and the blue bar indicates non-muscle-related gene sets.
2. Pathway gene set enrichment analysis. The top 10 pathways significantly enriched in Puro-bulk and Puro-clones with *FDR q*-values < 0.01 are shown. The red bar indicates muscle-related pathways, and the blue bar indicates non-muscle-related pathways.

**Supplementary Fig. S9 3D muscle tissues fabricated from six Puro-bulk *MYOD1*-hiPSCs and their sarcomere formation.**

a. Brightfield top-view images of 3D muscle tissues derived from six Puro-bulk *MYOD1*-hiPSCs at days 11 and 17. Scale bar, 500 μm.

b. IHC analysis of fabricated muscle tissues from six Puro-bulk *MYOD1*-hiPSCs for Titin and α-Actinin at day 17 of differentiation. Sarcomere formation was clearly observed in all muscle tissues. The nuclei were stained with Hoechst 33258. Scale bar, 50 µm.

**Supplementary Fig. S10 Time course gene expression analysis of 3D muscle tissues derived from hiPSCs.**

Time course gene expression analysis of the various myogenic markers, including *MYOD1* (total, transgene and endogenous), *MYF6*, *MYOG*, *MYH2*, *MYH7*, and *TMEM8C*, was performed using 3D muscle tissue derived from Puro-bulk 201B7-*MYOD1*-hiPSCs. n = 3 for days 11 and 13; n = 2 for days 15 and 17.

**Supplementary Table S1. Primer sequences and cycling conditions for qRT‒PCR and genomic qPCR**

**Supplementary Table S2. Antibodies used in this study**

**Supplementary Table S3. Primer sequences and cycling conditions for genomic PCR**

**Supplementary Table S4. Genes used for the clustering analysis**

**Supplementary Table S5: GO GSEA: Puro-bulk vs. G418-bulk (FDR q val < 0.05): GO GSEA: Puro-bulk**

**Supplementary Table S6: GO GSEA: Puro-bulk vs. G418-bulk (FDR q val < 0.05): GO GSEA: G418-bulk**

**Supplementary Table S7: Pathway GSEA: Puro-bulk vs. G418-bulk (FDR q val < 0.05): Pathway GSEA: Puro-bulk**

**Supplementary Table S8: Pathway GSEA: Puro-bulk vs. G418-bulk (FDR q val < 0.05): Pathway GSEA: G418-bulk**

**Supplementary Table S9: GO GSEA: Puro-bulk vs. Puro-clones (FDR q val < 0.05): GO GSEA: Puro-bulk**

**Supplementary Table S10: GO GSEA: Puro-bulk vs. Puro-clones (FDR q val < 0.05): GO GSEA: Puro-clones**

**Supplementary Table S11: Pathway GSEA: Puro-bulk vs. Puro-clones (FDR q val < 0.05): Pathway GSEA: Puro-bulk**

**Supplementary Table S12: Pathway GSEA: Puro-bulk vs. Puro-clones (FDR q val < 0.05): Pathway GSEA: Puro-clones**
