# Supplementary figures and images for: Simple and efficient differentiation of human iPSCs into contractible skeletal muscles for muscular disease modeling
Source: Sci Rep. 2023 May 25;13:8146. doi: 10.1038/s41598-023-34445-9 (PMC10213064; doi:10.1038/s41598-023-34445-9)

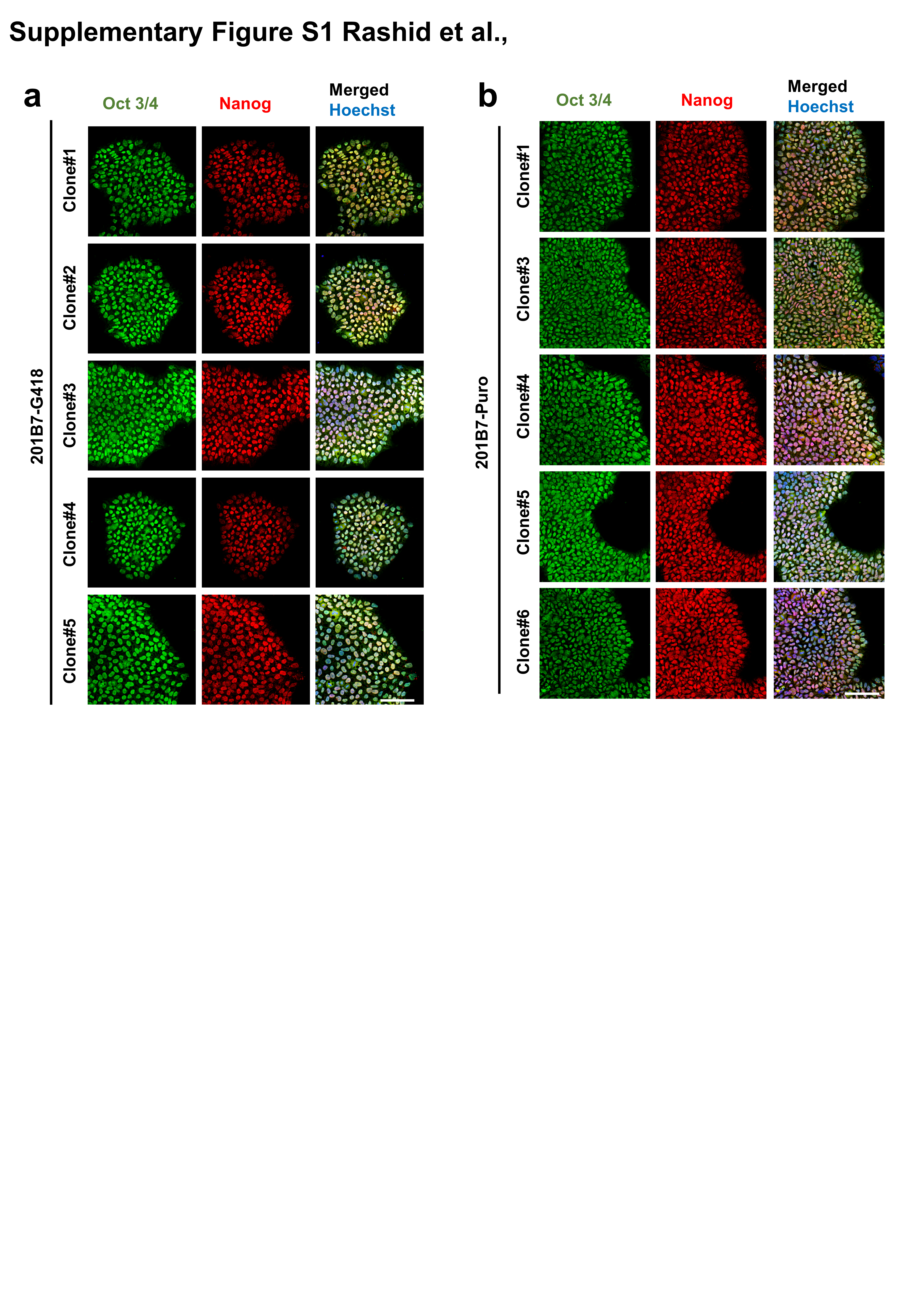

Supplement: Supplementary file 2 — Supplementary Figure S1. [file 41598_2023_34445_MOESM2_ESM.tif]

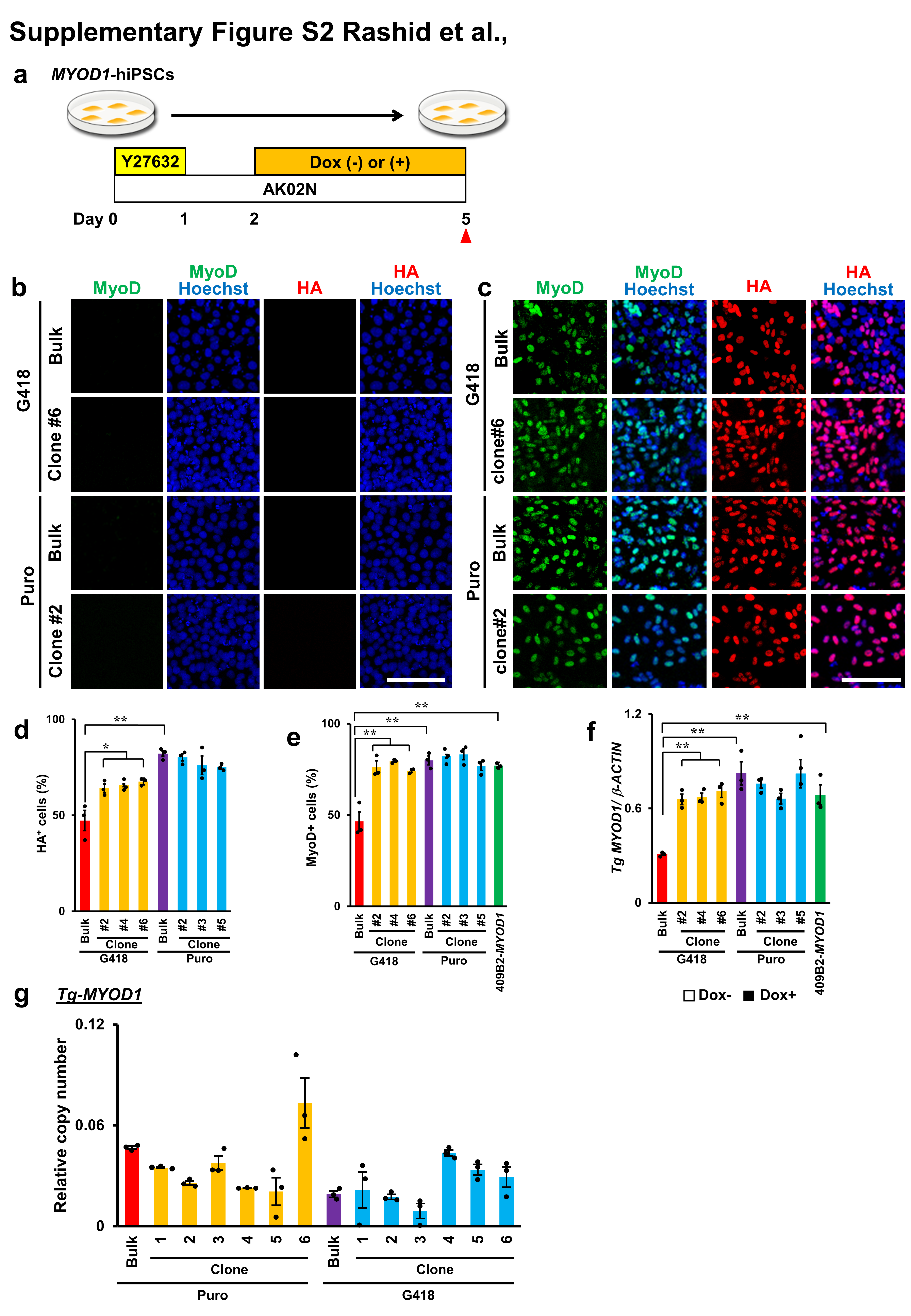

Supplement: Supplementary file 3 — Supplementary Figure S2. [file 41598_2023_34445_MOESM3_ESM.tif]

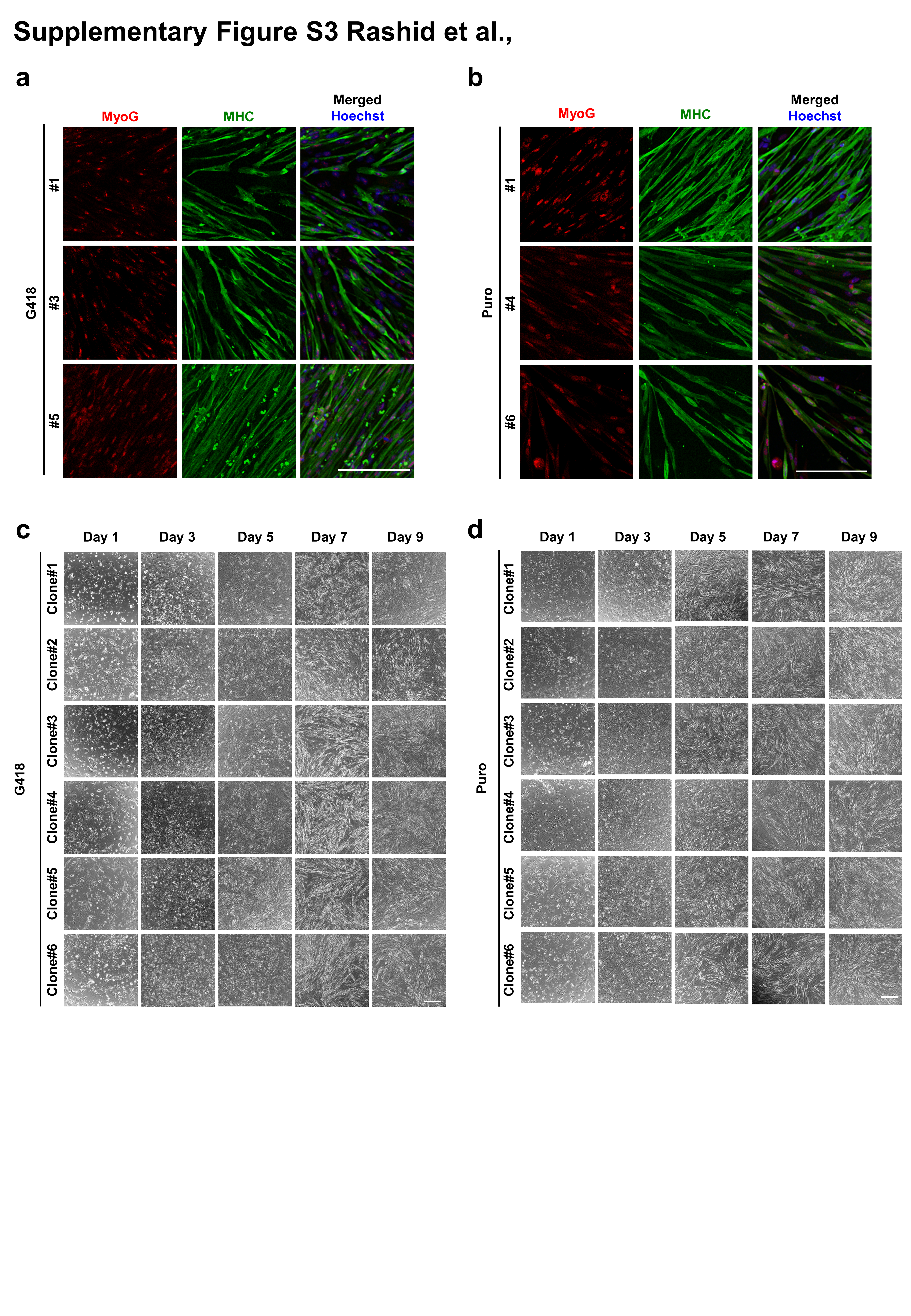

Supplement: Supplementary file 4 — Supplementary Figure S3. [file 41598_2023_34445_MOESM4_ESM.tif]

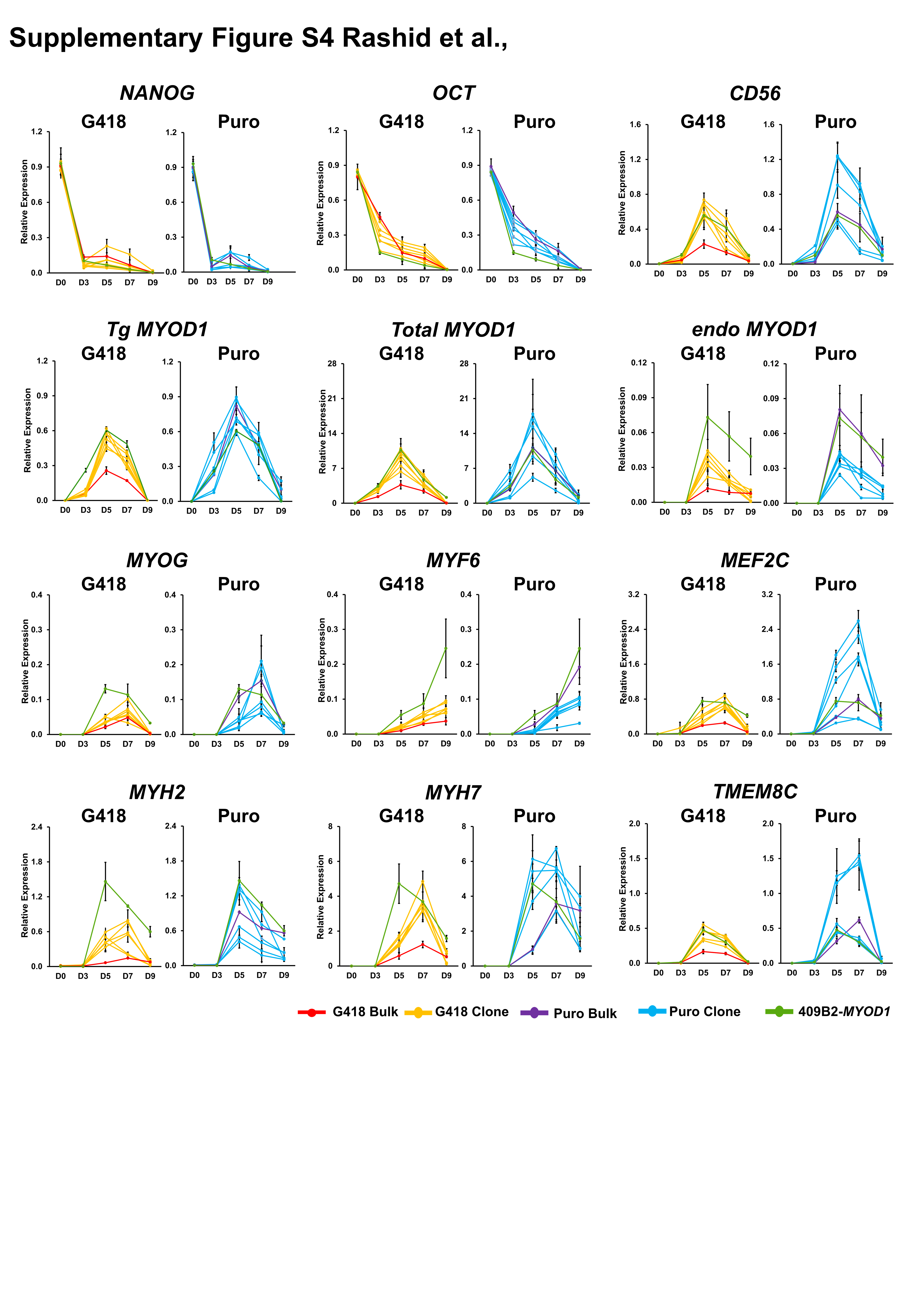

Supplement: Supplementary file 5 — Supplementary Figure S4. [file 41598_2023_34445_MOESM5_ESM.tif]

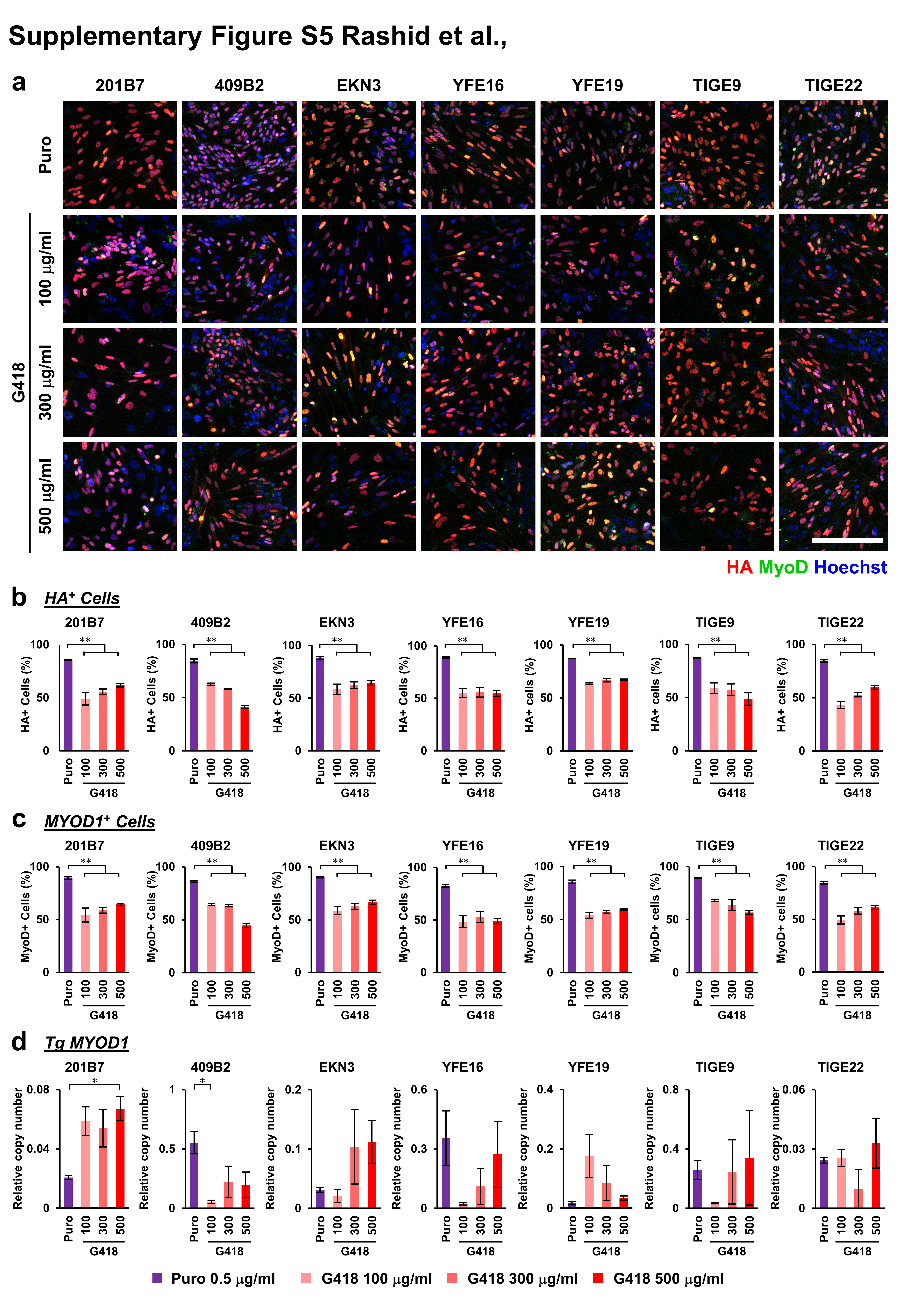

Supplement: Supplementary file 6 — Supplementary Figure S5. [file 41598_2023_34445_MOESM6_ESM.tif]

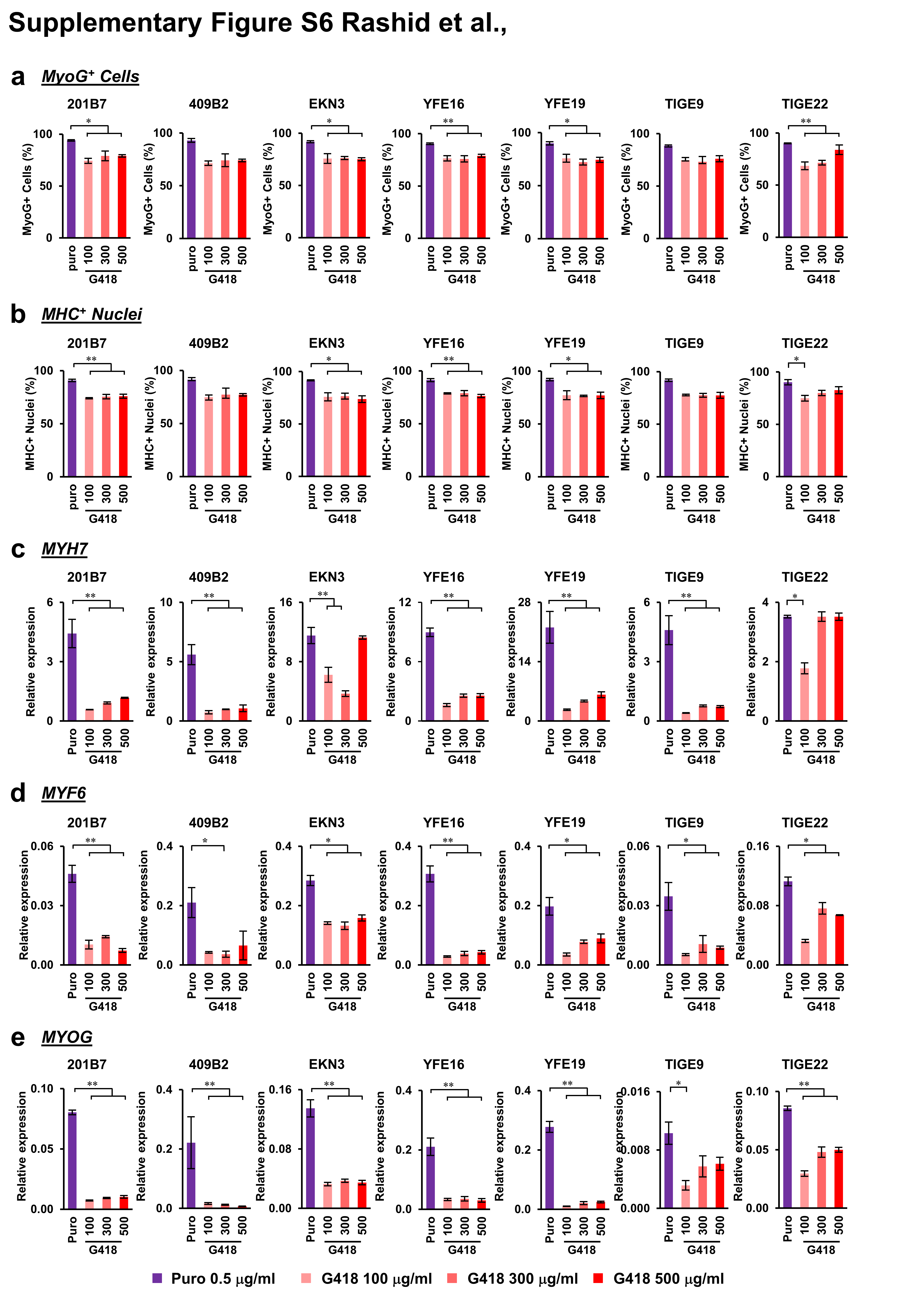

Supplement: Supplementary file 7 — Supplementary Figure S6. [file 41598_2023_34445_MOESM7_ESM.tif]

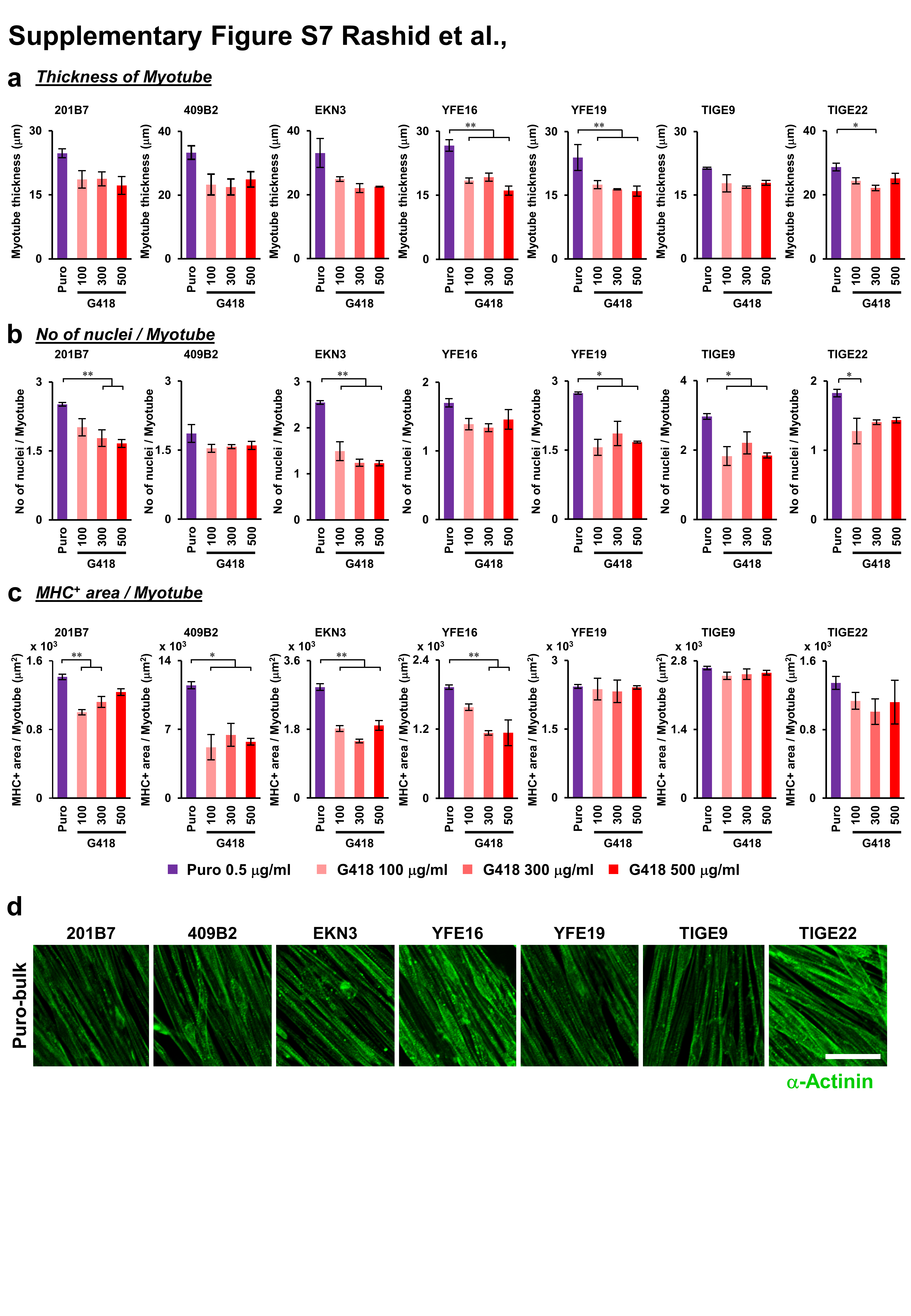

Supplement: Supplementary file 8 — Supplementary Figure S7. [file 41598_2023_34445_MOESM8_ESM.tif]

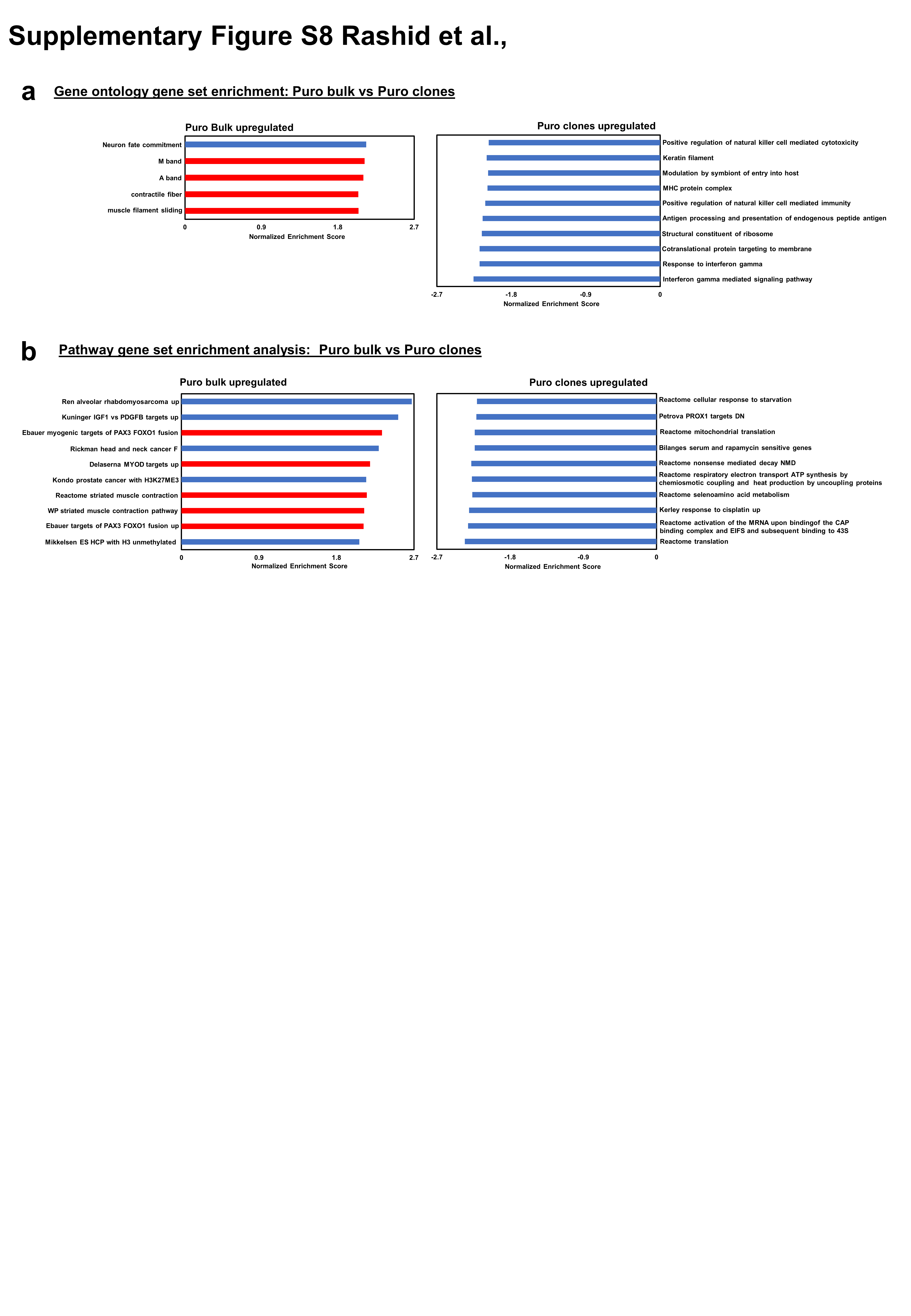

Supplement: Supplementary file 9 — Supplementary Figure S8. [file 41598_2023_34445_MOESM9_ESM.tif]

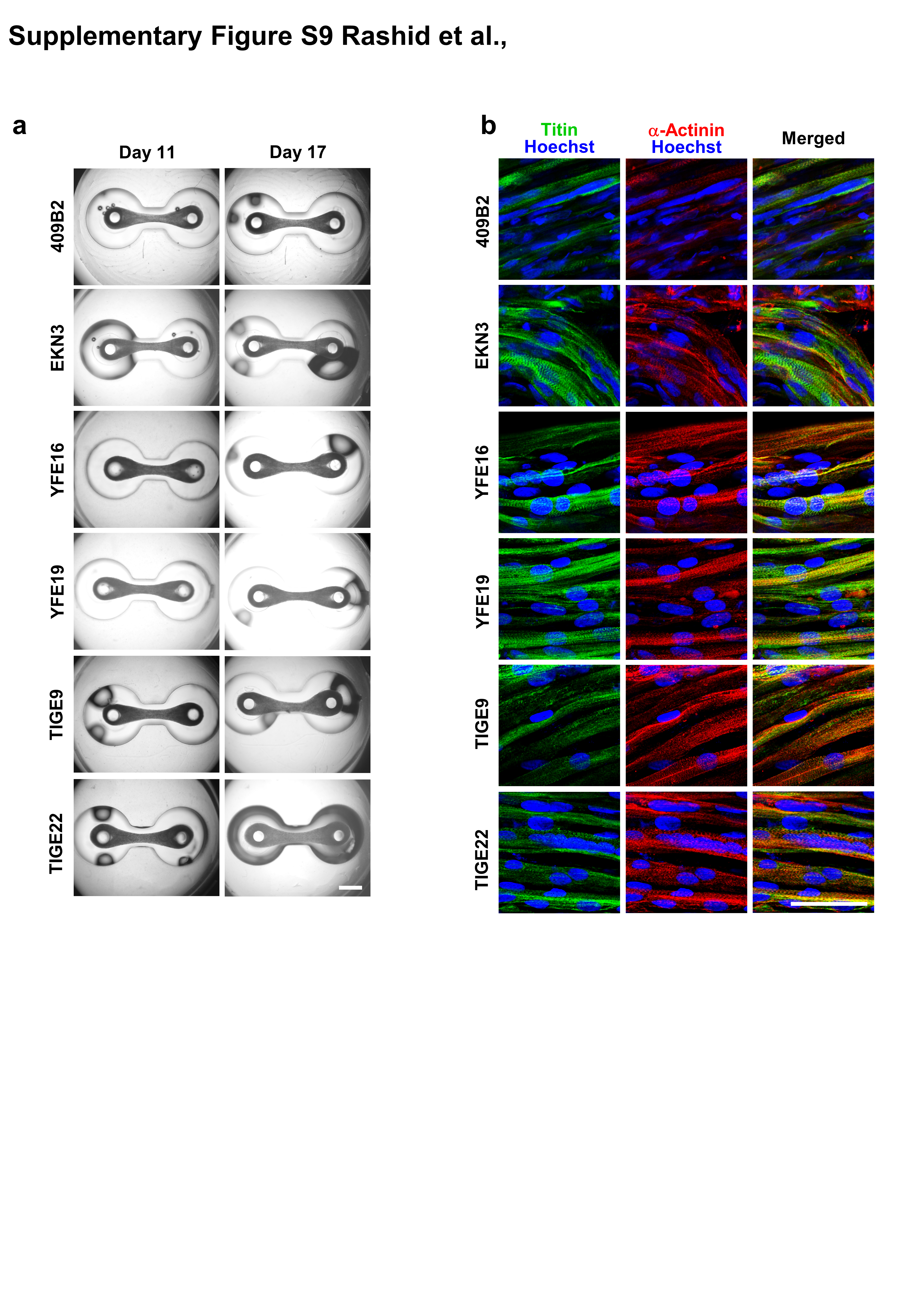

Supplement: Supplementary file 10 — Supplementary Figure S9. [file 41598_2023_34445_MOESM10_ESM.tif]

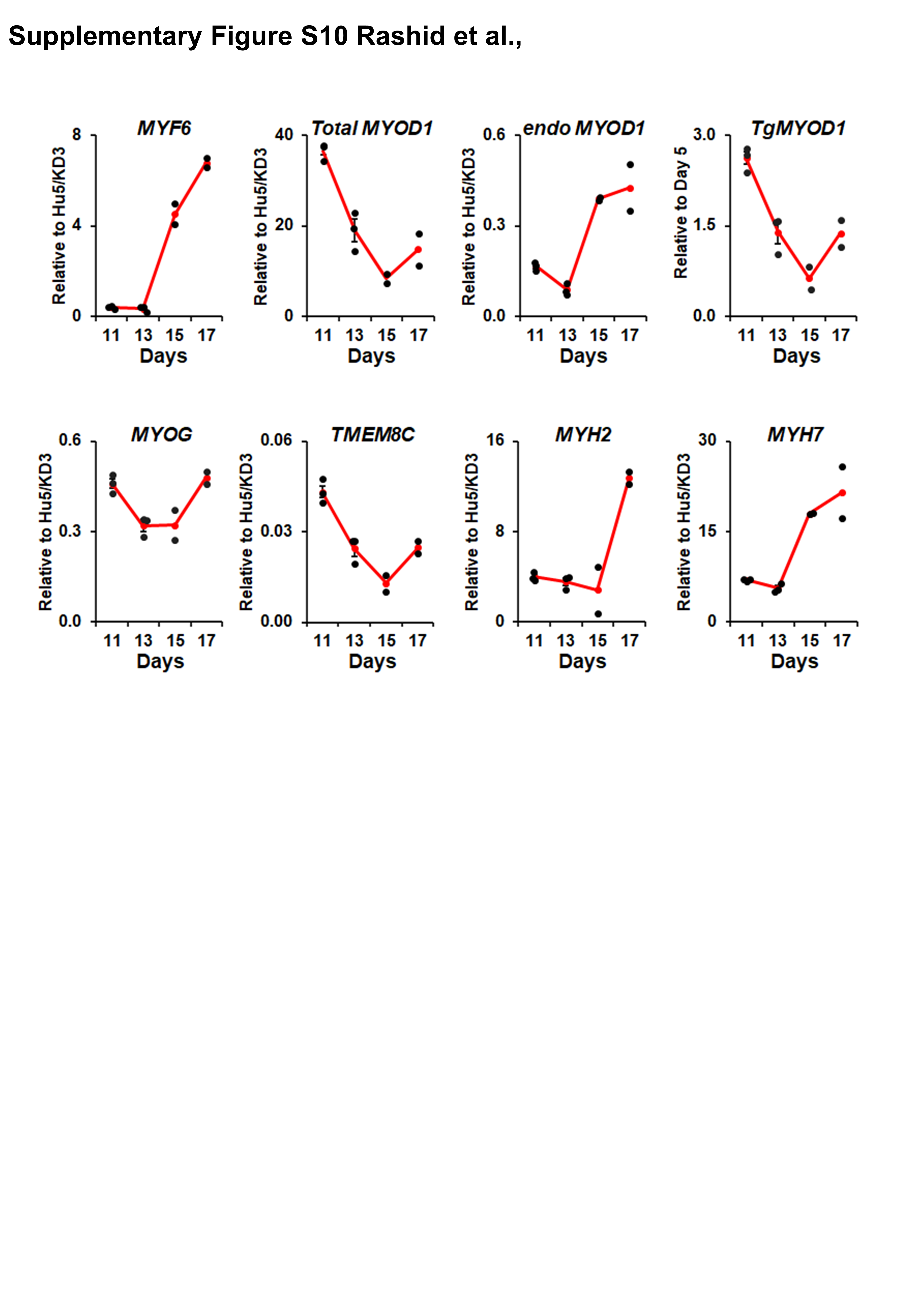

Supplement: Supplementary file 11 — Supplementary Figure S10. [file 41598_2023_34445_MOESM11_ESM.tif]
